# Advances in engineering organ-on-a-chip models of angiogenesis

**DOI:** 10.7150/thno.136769

**Published:** 2026-10-02

**Authors:** Yijun Tao, Ting Liu, Yi Zhang, Hongyan Yue, Huaiyi Zhang, Ke Lin, Chenxi Wu, Yasi Huang, Xiaran Liu, Xiaoli Wang, Fei Fang, Yang Shen

**Affiliations:** 1 Institute of Biomedical Engineering, West China School of Basic Medical Sciences & Forensic Medicine, Sichuan University, Chengdu 610041, China.; 2 Chengdu Rowic Organchip Biotechnology Co., Ltd., Chengdu, 610200, China.; 3 Department of Ultrasound, The First Affiliated Hospital of Chengdu Medical College, Chengdu 610500, China.; 4 Yale Cardiovascular Research Center, Yale School of Medicine, New Haven, CT 06511, USA.; 5 Institute of Panvascular Biology, JinFeng Laboratory, Chongqing, 401329, China.

**Keywords:** angiogenesis, microfluidics, bioprinting, endothelial mechanobiology, vascularized organoids

## Abstract

Angiogenesis plays a central role in embryonic development, tissue repair, tumor progression and cardiovascular remodeling. Because vascular growth involves a series of closely coordinated dynamic events, it is difficult for traditional *in vitro* and *in vivo* models to resolve the underlying mechanisms. Angiogenesis-on-a-chip (AoC) platforms provide microphysiological systems that can recapitulate key aspects of vascular growth *in vitro*, while enabling precise spatiotemporal control of the local microenvironment. These platforms combine microfluidic structures, three-dimensional hydrogels and relevant cell populations, so that biochemical gradients, mechanical cues and multicellular interactions can be regulated independently or in combination. When the relevant conditions are properly reproduced, endothelial sprouting, lumen formation and barrier function can be directly monitored and quantified. This review summarizes the recent progress of AoC design and discusses how these platforms can be used to study pathophysiological mechanisms, evaluate candidate therapeutic drugs and engineer vascularized tissues. The article also discusses persistent challenges, including platform standardization, characterization of the immune microenvironment, model validation and quantitative phenotypic analysis. By integrating engineering design with biological applications and an assessment of current limitations, this review provides a practical framework for advancing the application of AoC systems in vascular biology, therapeutic development and regenerative medicine.

## 1. Introduction

Angiogenesis refers to the formation of new blood vessels from pre-existing vasculature, which is a basic biological process that supports tissue development, repair, remodeling and disease progression 1. Under physiological conditions, angiogenesis is regulated in time and space, and is coordinated with the needs of development, repair or metabolism. Dysregulated angiogenesis, however, contributes to the pathogenesis of multiple diseases. For example, tumors release proangiogenic factors that sustain oxygen and nutrient delivery, increase vascular permeability, and facilitate invasion and metastasis 2. In chronic inflammatory diseases, newly formed and remodeled blood vessels can sustain leukocyte recruitment and accumulation, vascular leakage and sustained inflammatory signaling 3-4. Importantly, angiogenesis is a dynamic multistep process involving endothelial activation, extracellular matrix (ECM) remodeling, tip-stalk cell specification, sprout initiation and invasion, lumen formation, anastomosis, mural-cell recruitment, vascular remodeling, and finally perfusion or regression 5-6. Analyzing these coordinated events requires models capable of capturing not only cell morphology, but also spatiotemporal sequences such as sprouting, anastomosis, remodeling, perfusion and regression.

Conventional two-dimensional (2D) culture systems are simple to operate and reproducible, but they cannot reproduce three-dimensional extracellular-matrix architecture, tissue interfaces, matrix-mediated support, or flow-derived cues that govern vascular morphogenesis. Animal models retain whole-organism physiology, but their use is constrained by species differences, limited optical accessibility, cost, ethical considerations, and difficulty in independently controlling individual variables in mechanistic studies 7-8. These limitations are particularly prominent in the study of angiogenesis, because the behavior of endothelial cells is regulated by multiple factors such as ECM composition and stiffness, soluble-factor gradients, interstitial flow, wall shear stress (WSS), stromal- and mural-cell support, the immune microenvironment and tissue-specific metabolic needs 8-9. These limitations have promoted the development of alternative methods that can reproduce vascular biology more accurately.

Over the past decade, organ-on-a-chip (OoC) technology has emerged as a useful class of microphysiological platforms for reconstructing defined tissue microenvironments *in vitro*. OoC systems are engineered microfluidic platforms that integrate living cells or tissue constructs within controlled microenvironments. Instead of replicating entire organs, they emulate functional tissue units, enabling the controlled integration of fluid flow, mechanical stresses, and cell-cell interactions 10-11. For angiogenesis research, OoC systems can reconstruct key features of the microvascular niche, including 3D hydrogel matrices for cell-matrix signaling, controlled interstitial or luminal flow, spatially defined gradients of angiogenic or inflammatory factors, defined combinations of endothelial, mural, stromal, immune, and parenchymal cells, and optical access for real-time imaging of sprouting, lumen formation, perfusion, permeability, and regression 12-13. Consequently, in the context of angiogenesis research,* in vitro* chip platforms specifically designed for angiogenesis studies (termed angiogenesis-on-a-chip, AoC) are being increasingly employed to investigate mechanisms of vessel formation, evaluate pro- or antiangiogenic interventions, and engineer vascularized tissues for regenerative medicine applications.

To clarify the scope of this review, several related vascular terms are distinguished in Table 1. This distinction is essential because the presence of endothelial cells, platelet endothelial cell adhesion molecule-1 (PECAM-1, also known as CD31)-positive vessel-like structures, or endothelialized channels does not by itself demonstrate angiogenic vessel formation. Accordingly, this review does not aim to provide a general overview of all vascularized organoids, endothelialized channels, or vascularized OoC systems. Instead, we use the term AoC to refer specifically to chip-based microphysiological systems in which angiogenic behavior is the primary process being recapitulated, perturbed, or quantitatively analyzed. Vascularized organoids, self-assembled vascular networks, endothelialized microchannels, and bioprinted vascular structures are discussed here only when they offer direct insights into angiogenic sprouting or its associated remodeling, anastomosis, perfusion, barrier formation, and maturation. This distinction is essential because vascular support strategies and angiogenesis models, while related, are not interchangeable. By making this delineation, we position AoC as a distinct experimental framework in which engineering controllability is translated into mechanistic interpretability and application-oriented readouts of vascular function.

This review discusses the current design principles, biological foundations, and translational applications of AoC systems. We first outline the core biological principles that define angiogenesis-directed modeling. We then compare major engineering strategies employed in AoC, including vascular patterning, self-assembled microvascular networks, bioprinting, and stem-cell-based approaches. We next examine how biomaterials, ECM properties, flow control, and cellular composition collectively determine AoC performance. Finally, we highlight representative applications in disease modeling, antiangiogenic and proangiogenic drug evaluation, vascular-targeted delivery, imaging-compatible phenotyping, and vascularized tissue engineering, and discuss remaining challenges in standardization, patient-specific modeling, functional validation, correlation with *in vivo* outcomes, and computational or artificial intelligence-driven analysis.

## 2. Physiological and Pathological Mechanisms of Angiogenesis

To construct and study functional vascular networks *in vitro*, it is essential to first define the biological principles governing angiogenesis and subsequently translate these principles into well-defined engineering objectives. Unlike vasculogenesis, which denotes de novo vessel formation, angiogenesis denotes the formation or expansion of vessels from pre-existing vasculature through sprouting, intussusception, and related vessel-derived remodeling modes 1, 18. Under physiological conditions, angiogenesis functions not as a unidirectional cascade of endothelial sprouting, but rather as a tightly regulated, context-dependent program of vascular remodeling.

During embryonic development and organ maturation, nascent vessels not only supply oxygen and nutrients to metabolically active tissues but also provide endothelial angiocrine cues that orchestrate tissue patterning, maintenance of progenitor-cell niches, and establishment of specialized barriers 19-20 (Figure 1A). In wound healing and tissue regeneration, angiogenesis is transiently reactivated in response to injury: inflammatory signals and provisional extracellular matrix remodeling stimulate endothelial cell activation, invasion, and lumen formation, while the newly formed vessels facilitate immune-cell trafficking, clearance of cellular debris, fibroblast migration, granulation tissue formation, and re-epithelialization 21-22. In ischemic tissues, reduced oxygen tension activates hypoxia-inducible factor (HIF)-dependent programs and increases vascular endothelial growth factor (VEGF) signaling 2, 23. Spatial VEGF cues, together with Delta-like ligand 4 (DLL4)-Notch signaling, then regulate tip–stalk dynamics and sprout extension 2, 24.

When these mechanisms become persistent, excessive, or spatially disorganized, angiogenesis can contribute to disease-promoting vascular dysfunction. In solid tumors, chronic hypoxia, oncogenic signaling, inflammation, and aberrant matrix remodeling sustain VEGF-driven neovascularization and generate tortuous, immature, and highly permeable vessels. This abnormal vasculature supports tumor growth, contributes to elevated interstitial fluid pressure, facilitates invasion and metastasis, and can impair drug delivery 25. In proliferative diabetic retinopathy, pathological retinal neovascularization is associated with endothelial dysfunction and heterogeneous immune-cell responses. Recent profiling of patient-derived specimens identified distinct disease patterns associated with immune defense or endothelial mitochondrial dysfunction, indicating that immune activation should not be treated as uniformly proangiogenic 26. In chronic inflammatory microenvironments, vascular activation and remodeling can sustain endothelial activation and leukocyte transmigration, reinforcing feedback among vascular leakage, inflammatory mediators, and immune-cell recruitment 27-28.

Angiogenesis involves regulatory modules that overlap in space and time, and these modules do not constitute a strict linear cascade (Figure 1B). Their relative contributions depend on the tissue context, oxygen tension, inflammatory status, ECM composition and hemodynamic cues. Endothelial cell activation is accompanied by matrix metalloproteinase (MMP)-dependent remodeling of the vascular basement membrane and surrounding ECM. Adhesive ligands, mechanical resistance, and matrix-bound growth factors provided by the ECM influence endothelial invasion, lumen formation, and vascular stability 29-30. Endothelial junctions also undergo dynamic remodeling. Vascular endothelial growth factor receptor 2 (VEGFR2) signaling and local biochemical and mechanical cues jointly regulate vascular endothelial cadherin (VE-cadherin)-associated adherens junctions and tight-junction components, including ZO-1, claudin-5, and occludin 31. ZO-1 also regulates the endothelial stress response during angiogenesis 32. These processes maintain a balance between the plasticity required for sprouting and the adhesion between cells required for collective migration. In response to local cues, endothelial cells dynamically adopt tip-like or stalk-like states. VEGF-VEGFR2 signaling supports tip-cell polarization, filopodial extension and gradient sensing; DLL4-Notch-mediated lateral inhibition limits excessive tip-cell selection, supporting stalk-cell specification and coordinated sprout extension 2, 33-34. Nascent vessels must lumenize, anastomose, and stabilize to support vascular function. These events overlap each other and do not follow a fixed order. Anastomosis between sprouts or between sprouts and pre-existing vessels establishes continuous lumens, thereby enabling perfusion. Angiopoietin-1 (Ang-1)/Tie2 signaling contributes to vascular maturation and barrier stability, whereas platelet-derived growth factor-BB (PDGF-BB)/platelet-derived growth factor receptor β (PDGFRβ) signaling promotes mural-cell recruitment; basement-membrane deposition and endothelial-mural cell interactions also contribute to these processes 31, 35-36. After the perfusion is established, the flow-dependent mechanical cues interact with the endothelial metabolism to shape the subsequent remodeling. WSS and interstitial flow regulate endothelial organization, junctional stability and mechanotransduction 37. Glycolysis, fatty acid oxidation, mitochondrial function and autophagy affect migration, proliferation, stress adaptation and endothelial-state heterogeneity 38-39. Growth factors or vascular morphology alone cannot explain angiogenesis, which comes from the coordinated response of cells to the changing microenvironment.

These biological principles directly inform AoC design and validation. Vascular growth depends on interaction among endothelial, mural, stromal and immune cells, and is also influenced by ECM properties, soluble-factor gradients and mechanical cues. Biomimetic AoC systems should incorporate the interactions most relevant to their intended application, and validation should not be limited to morphological readouts such as sprout length, branch number and network density 40. Although these structural readouts remain useful for describing early morphogenesis, they are insufficient to establish whether an engineered vascular network is functionally meaningful. Therefore, AoC platforms should reproduce key regulatory features of the angiogenic microenvironment, including spatially defined biochemical gradients, degradable and mechanically tunable matrices, controllable interstitial and luminal flow, and multicellular configurations that support vessel stabilization. Evidence on metabolic regulation and autophagy identifies additional mechanistic readouts 38-39. More broadly, AoC validation should combine morphological readouts with functional vascular endpoints, such as lumen formation, anastomosis, perfusability, permeability, mural-cell coverage, inflammatory activation, shear responsiveness, and long-term vascular remodeling 13, 40. In this sense, AoC platforms are valuable not merely because they reproduce visible endothelial sprouting, but because they allow individual angiogenic variables to be introduced, isolated, and recombined under controlled conditions, thereby dissecting regulatory mechanisms that are difficult to resolve *in vivo*.

## 3. Engineering Strategies for AoC

For practical purposes, recent AoC development principles can be synthesized into three linked questions: which biological processes and functions must be reproduced, how the model should be constructed and operated, and how its fitness for purpose should be demonstrated 41 (Figure 2A). Design, simulation, prototyping, and testing are iterative.

First, investigators define the biological question and context of use (CoU) by specifying the angiogenic process, experimental conditions, and the decision context the model should support 42. They then specify what the model should reproduce, how these features will be measured, and what results will be considered acceptable. For example, a sprouting model may quantify sprout initiation and extension 13. Endothelial viability should be monitored in parallel, while vehicle controls and reference pro- or antiangiogenic treatments can be used to define acceptable assay performance 43. These choices define biological requirements, including the endothelial phenotype, supporting cells, ECM, soluble cues, and culture and maturation conditions 13, 42. They also define the engineering requirements, including chip geometry, fluidic control, perfusion connections, imaging and sampling access, and sensor performance 42, 44.

Second, the vascular construction strategy and chip layout are selected together 45. Patterning defines vessel geometry, including parent vessels for controlled sprouting. Self-assembly allows endothelial and supporting cells to form microvascular networks within a matrix, which may subsequently undergo sprouting or remodeling 13. Bioprinting positions cells and matrix during fabrication and can be combined with either strategy 8, 13. Material and fabrication choices should be coordinated with chip architecture, cell populations, and ECM requirements 8, 44. These materials and methods should support cell culture and imaging, allow appropriate gas and solute transport, achieve repeatable manufacturing, and reduce unexpected compound loss 8, 43. The platform architecture determines the order of assembly, sterilization, surface coating and cell/ECM loading, but there is no fixed order applicable to all systems 8, 44. Computational fluid dynamics (CFD) and transport simulation can be used to compare different layouts and estimate the distribution of flow, WSS and oxygen, nutrients or drugs 15, 44. These predictions should be verified by prototype measurement, and the geometric structure or perfusion conditions should be adjusted if necessary 15. When conditions permit, the sealing integrity, fluidic connections and flow characteristics should be confirmed before biological loading 41, 43. In open systems or bioprinting systems, cells and ECM materials may need to be introduced during the manufacturing process or before final assembly 8. The selected vascular construction strategy determines how tissue formation, maturation and perfusion activation are coordinated 13, 41. After the biological loading is completed, the fluid conditions should be re-evaluated, and the operating parameters should be optimized according to the predefined readouts 42, 44.

Third, the integrated model needs to be evaluated according to the predefined CoU. Technical validation covers device size, sealing integrity, bubble control, flow and pressure control, compound recovery, sensor calibration and inter-chip consistency 42-43. Biological validation confirms whether the model reproduces the vascular characteristics required for its intended application, including endothelial viability and phenotype, lumen continuity, perfusion, barrier integrity, mural-cell coverage, and response to reference stimuli or treatment 13, 42. Repeatability should be assessed within experiments, whereas reproducibility should be evaluated across experiments, donors, device batches, and laboratories where relevant 43-44. Any unmet acceptance criteria should prompt the inspection of biological design, device design or operating conditions to identify the source of deviation and then revise and retest the model.

To clarify the methodological diversity underlying AoC construction, Table 2 compares vascular patterning, self-assembled vascular networks, and 3D bioprinting/stem-cell co-differentiation in terms of construction principle, angiogenesis relevance, validation endpoints, strengths, limitations, and best-fit applications.

Collectively, this comparison highlights that the three strategies are not interchangeable routes to the same endpoint. Vascular patterning is particularly useful when experimental objectives demand precise control over vascular geometry, hemodynamic flow, biochemical gradients, and the timing of perturbation. Self-assembly is better suited to studies prioritizing emergent capillary morphogenesis, multicellular remodeling, and organ-specific vascular maturation. 3D bioprinting and stem-cell co-differentiation are complementary options when spatially programmable, scalable, or stem-cell-derived vascularized constructs are required. Therefore, the choice of engineering strategy should be dictated by the biological question and by the level of control, self-organization, perfusion, and maturation required. Across all engineering strategies, the mechanism of vascular formation should be distinguished from functional maturation. Angiogenesis requires direct evidence that new vascular structures arise from a pre-existing vessel, whereas lumenization, anastomosis, perfusion, permeability, vascular stability, and remodeling characterize vascular connectivity, function, or post-formation maturation.

### 3.1 Vascular Patterning

Vascular patterning technology enables the construction of perfusable, vessel-like luminal structures at predefined locations, making it particularly well suited for AoC models requiring rigorous control over vascular geometry, endothelial-matrix interfaces, flow direction, and angiogenic cue distribution. This is typically achieved using microfabricated channels, sacrificial templates, or topographical guidance cues, which are subsequently lined with endothelial cells and positioned adjacent to three-dimensional hydrogel compartments. In this context, a patterned endothelialized channel should be considered a model of angiogenesis only when spatially or temporally resolved evidence demonstrates that new vascular growth originates from the pre-existing channel, for example through endothelial sprouting and matrix invasion 9, 13, 17. Lumen continuity and anastomosis characterize structural integration, whereas perfusion and post-formation remodeling reflect vascular function or subsequent adaptation; these endpoints do not, by themselves, establish angiogenesis 13, 50, 58. Predefined channels provide control over lumen dimensions and spacing, flow direction, the endothelial–matrix interface, and local mechanical conditions, although the resulting biological response still requires experimental validation. Representative patterned systems have used parallel channels, microposts, and hydrogel compartments to constrain the direction of vascular outgrowth 59. With advances in rapid prototyping and open-access microfluidic fabrication technologies, the construction of patterned devices has become more straightforward and is now widely used in transport studies and drug testing involving perfusable vascular constructs 60-61. Current AoC platforms combine patterned vasculature with controlled shear stress, interstitial flow, and soluble concentration gradients, providing a defined experimental foundation for investigating endothelial sprouting, vascular stabilization, barrier formation, and spatial endothelial heterogeneity 39, 46. A representative example is the nanoporous hydrogel-based angiogenesis model reported by Mote *et al*., in which a micromolded endothelialized tube embedded within a three-dimensional hydrogel matrix was connected to a perfusion system. This design enabled simultaneous control of luminal flow-induced shear stress and chemokine gradients, allowing angiogenic sprouting from a patterned parent vessel to be analyzed under defined mechanical and biochemical conditions 46 (Figure 2B-i).

The main advantage of vascular patterning is experimental control, particularly when isolating the effects of geometry, matrix properties, biochemical gradients, or WSS on endothelial behavior. Defined geometry and perfusion conditions make these systems useful for mechanobiology, vascular barrier analysis, and drug screening. Multiphoton-guided hydrogel ablation can extend this control to complex, organ-specific microvascular architectures that can be endothelialized and perfused 47. Accessible fabrication approaches, including open microfluidics and benchtop xurography, reduce the need for specialized cleanroom facilities 60-61. Vessel-chip models have been used to examine how matrix stiffness modifies endothelial responses to shear stress 62. Patterned platforms also support scalable antiangiogenic screening. Anchor-IMPACT combines spatial hydrogel confinement, injection-molded arrays, and image-based quantification to compare compounds under standardized, high-throughput conditions 48.

Patterned systems impose geometric constraints that can limit spontaneous capillary self-organization, irregular anastomosis, adaptive pruning, and changes in vascular hierarchy during maturation or regression. Bubble formation, hydrogel leakage, incomplete endothelialization, heterogeneous endothelial–matrix interfaces, and compound sorption by polydimethylsiloxane (PDMS) can also alter local flow or solute exposure. Computational modeling can help quantify transport and mechanical conditions but does not itself eliminate these experimental limitations. For example, AngioMT, a MATLAB-based two-dimensional image-to-physics computational tool, predicts oxygen transport in vascularized microphysiological systems 63. Comparisons of perfusion methods can reveal differences in endothelial transcriptional state and function 64. In a multilayer chip containing initially dispersed ECs, higher modeled shear was associated with less extensive self-assembled network formation, and prolonged high-shear exposure promoted network degradation 65. Because the initial network formed de novo, this system is more appropriately classified as vasculogenesis-like self-assembly than as patterned-vessel angiogenesis.

More broadly, related microfluidic vascularization principles have also been applied in vascularized urethra- and kidney-on-a-chip systems for tissue repair and disease modeling 66-67. Therefore, patterned AoC models should be evaluated not only by structural endpoints, such as sprout length, branch number, and network density, but also by functional readouts, including lumen continuity, dextran or bead perfusion, permeability, endothelial junction organization, mural-cell coverage, and shear responsiveness. Overall, vascular patterning technology is most valuable when vascular geometry, flow parameters, and mechanical stimulation need precise definition. When closer recapitulation of spontaneous and dynamic *in vivo* vascular networks is required, patterned systems are best suited for controlled screening or mechanistic perturbation studies, either alone or combined with self-assembly strategies.

### 3.2 Self-Assembled Vascular Networks

Self-assembled vascular networks are generated not by endothelializing predesigned lumens but by allowing initially dispersed ECs, often together with stromal or mural cells, to organize within a 3D ECM under the coordinated regulation of biochemical signals, matrix properties, cell–cell interactions, and fluid-mechanical cues 13, 40. The ECs migrate, establish intercellular contacts, align, form lumens, and organize de novo into interconnected microvessels 13. Because no pre-existing vascular structure serves as the source of these vessels at the beginning of culture, this initial process is more accurately described as vasculogenesis-like self-assembly rather than angiogenesis 13, 17. Compared with vascular patterning, self-assembly is particularly well suited for investigating emergent capillary morphogenesis, multicellular coordination, capillary-scale branching, network interconnection, dynamic network formation, and subsequent post-formation remodeling. However, this biological flexibility is accompanied by reduced control over lumen geometry, local shear-stress distribution, and final network topology 13, 40. Once established, the resulting network may serve as pre-existing vasculature for investigating subsequent angiogenic growth, but angiogenesis should be assigned only when vessel-derived new growth, such as sprouting from the established network, is directly demonstrated 9, 13.

Because de novo self-assembly does not by itself ensure controlled vascular access, directional perfusion, or integration with larger vessels, a major engineering objective is to connect the emergent capillary bed to predefined inflow and outflow boundaries. The model developed by Hirth *et al*. illustrates how this integration can be achieved without conflating the underlying biological processes. Arterial ECs, venous ECs, and pericytes first underwent vasculogenesis-like self-assembly within the central fibrin hydrogel to form a capillary network 50 (Figure 2B-ii). ECs lining the arteriole- and venule-like side channels subsequently generated angiogenic sprouts into the central hydrogel, which anastomosed with the self-assembled capillaries to establish an interconnected and perfusable arteriole–capillary–venule network. VEGF was supplied during the initial culture period, but no VEGF gradient was imposed across the side channels; instead, interstitial flow promoted side-channel sprouting and anastomotic integration. Subsequent permeability analysis and leukocyte trafficking assays evaluated vascular function and downstream applications rather than the origin of the central capillary bed. Chen *et al*. extended the same engineering principle across vascular scales by combining patterned arteriole- and venule-like channels with a self-assembled capillary compartment 58. Subsequent anastomosis and directional perfusion established hierarchical microcirculation suitable for permeability measurements and studies of circulating-cell arrest and extravasation. Together, these platforms show that predefined perfusion boundaries can preserve emergent capillary morphogenesis while adding vascular hierarchy, flow directionality, and functional access. Anastomosis and perfusion demonstrate structural and functional integration but do not retrospectively redefine the initial capillary-bed formation as angiogenesis 50, 58.

The lack of a predefined vascular template permits spontaneous capillary morphogenesis but also makes the resulting network architecture less predictable than in patterned systems. Accordingly, device-to-device differences may arise in network density, connectivity, vessel diameter, local shear stress, nutrient transport, matrix remodeling, perfusability, and permeability. This variability is especially consequential in tumor models, in which the vascular phenotype itself influences drug transport and therapeutic response. For example, Ahn *et al*. generated self-assembled intratumoral vessels that exhibited peritumoral angiogenic sprouting and vascular leakage and used the platform to investigate interstitial-flow-dependent blood- and lymphatic-vessel growth and responses to the antiangiogenic agent axitinib 68. Current platforms therefore increasingly employ “soft-guidance” strategies, defined here as biochemical, mechanical, cellular, or boundary cues that bias where, when, or in which direction vessels develop without prescribing the geometry of every lumen. The appropriate guidance strategy depends on the biological question. When the objective is to investigate biochemical–mechanical regulation of lymphangiogenesis, vascular endothelial growth factor C (VEGF-C) concentration, spatial gradients, and interstitial flow can be varied independently or in combination to dissect their effects on lymphatic sprouting and network formation 69. When organ-specific vascular maturation or barrier function is the primary endpoint, tissue-relevant supporting cells and perfusion cues become more important. A self-assembling brain vessel-on-chip containing human induced pluripotent stem cell (iPSC)-derived ECs and astrocytes together with human brain vascular pericytes showed that cyclic adenosine monophosphate signaling or continuous perfusion improved network organization and reduced vascular permeability 51. These improvements did not establish a fully mature blood–brain barrier phenotype. In a separate hepatic model, self-assembled perfusable hepatic sinusoid networks were incorporated into liver lobule-like units containing hepatic tissue 52. Thus, self-assembly is not a single-purpose angiogenesis assay but a flexible vascularization strategy for investigating capillary morphogenesis, organ-specific vascular function, tumor–vascular interactions, drug transport, immune-cell trafficking, and lymphatic–vascular interactions.

Because self-assembled networks can be used to study both initial de novo network formation and subsequent angiogenic growth, their evaluation should follow the process being examined. For de novo self-assembly, studies should report how ECs and supporting cells were initially positioned, their sources, ECM composition, applied biochemical and mechanical cues, and time-dependent changes in network density, branch length, and lumen formation 13, 40. When an established network is used to model subsequent angiogenesis, time-resolved imaging or spatially resolved analysis should demonstrate that new vascular structures originate from that network 9, 13, 17. Such analyses can also reveal how interacting cell types are organized during sprouting. For example, Sano *et al*. showed that mesenchymal stromal cells selectively localized around the protrusions and roots of angiogenic sprouts 70. Once the network has formed, connectivity, lumen continuity, and anastomosis indicate structural integration. Perfusability and permeability indicate vascular function, whereas mural-cell coverage, basement-membrane deposition, long-term stability, and post-formation remodeling indicate maturation and subsequent vascular adaptation.

### 3.3 3D Bioprinting and Stem-Cell Co-Differentiation

3D bioprinting and stem-cell co-differentiation represent two complementary but mechanistically distinct strategies for generating vascularized AoC and organoid-on-chip systems. Bioprinting provides spatial control over cells, extracellular matrix, bioactive cues, and, in some designs, perfusable channel templates, whereas stem-cell co-differentiation introduces developmentally related parenchymal, endothelial, and stromal lineages within the same tissue construct. Therefore, bioprinted vascular scaffolds, co-differentiated vascular organoids, and vascularized organoid-on-chip systems should not be regarded solely as angiogenesis models. Their relevance to AoC depends on their ability to support controlled endothelial cell sprouting, vascular network formation, perfusability, remodeling, or functional vascular maturation within a well-defined microenvironment on the chip. When integrated with perfusion platforms, 3D bioprinting enables precise placement of cells and matrices together with the fabrication of channel templates, but it does not eliminate the need for subsequent endothelialization, vascular maturation, and functional validation. Recent bioprinting approaches relevant to AoC have emphasized spatially controlled and reproducible fabrication together with perfusion integration. A representative example is the multimaterial, sacrificial bioprinting platform developed by Maggiotto *et al*., in which cell-laden GelMA and Pluronic F127 were co-printed within a custom bioreactor; removal of the sacrificial ink generated a perfusable channel that was subsequently endothelialized and maintained under flow for up to 21 days 71 (Figure 2B-iii). The model supported endothelial-barrier formation and microvascular outgrowth from the perfused channel toward neuroblastoma aggregates, thereby coupling spatially controlled tissue placement with perfusion and angiogenic remodeling. This strategy is particularly useful when organ-specific architecture, multicellular spatial organization, or reproducible tissue placement is required. However, printing accuracy does not necessarily translate into vascular function, because printed channels or cell-laden bioinks must still support endothelial viability, lumen continuity, stable perfusion, barrier integrity, and long-term tissue maturation.

In parallel, stem-cell co-differentiation and engineered vascular niche strategies have advanced the generation of vascularized organoid systems. Recent work has shown that co-development of mesodermal and endodermal lineages can promote organotypic vascularization in lung and gut organoids 72, while engineered endothelial niches can improve vascularization, multilineage maturation, and the emergence of renin-expressing cells in human kidney organoids 73. These studies provide developmental principles relevant to vascularized AoC design but remain distinct from chip-based angiogenesis models unless chip integration and vessel-derived growth are demonstrated. A related on-chip example used iPSC-derived cardiac microtissues whose preformed microvessels anastomosed with an external vascular network in fibrin, enabling perfusion 55. Such platforms illustrate how stem-cell-derived parenchymal and vascular compartments can be combined with microfluidic control, but they also highlight persistent challenges related to iPSC immaturity, batch-to-batch variability, incomplete mural-cell maturation, and limited control over capillary-scale architecture.

The choice of fabrication method should therefore be matched to the biological question. Multi-nozzle extrusion and drop-on-demand printing are useful for scalable placement of hydrogels and cell populations, whereas light-based methods such as digital light processing (DLP) offer higher spatial resolution but may impose phototoxicity, restricted bioink chemistry, and trade-offs between resolution and cell viability. Earlier DLP-based bioprinting studies illustrated how light-based printing can spatially organize osteogenic and vascular compartments in hydrogel constructs 74, although these constructs should not be considered chip-based AoC models *per se*. In addition, recent reviews of vascularized organoids, vascularized organoid-on-chip models, and 3D-printing-based OoC emphasize that co-differentiation, organoid-on-chip integration, and 3D bioprinting should be considered complementary vascularization strategies rather than a single uniform approach 14.

For practical use, the central issue is not whether vascularized constructs can be printed or co-differentiated, but whether they remain perfusable, mature, and functionally interpretable over time. Shear stress during extrusion, light dose during photocrosslinking, and bioink stiffness can alter endothelial phenotype, stem-cell differentiation, and tissue maturation. In addition, relatively large, printed channels may be required to maintain stable perfusion, which can oversimplify capillary-scale microvascular geometry. Therefore, bioprinted and co-differentiated vascularized AoC models should be validated using functional endpoints, including lumen continuity, perfusion stability, vascular permeability, endothelial junction organization, mural-cell coverage, matrix deposition, tissue-specific maturation markers, and response to drugs or angiogenic cues. More broadly, OoC-based 3D angiogenesis assays have been applied to phenotypic screening, including large-scale kinase inhibitor screening, highlighting the translational potential of engineered vascular models beyond fabrication itself 49. Overall, the value of 3D bioprinting and stem-cell co-differentiation lies in integrating spatial biofabrication with developmental vascular biology, but broader adoption will require improved control over vascular maturation, perfusion stability, capillary-scale organization, and batch-to-batch consistency.

## 4. Key Components and Design Elements of AoC

The performance of an AoC platform is determined by the coordinated design of the device substrate, 3D ECM scaffold, physical cues, biological components, and validation readouts, rather than by any single element alone (Figure 3). These elements define whether a platform merely supports endothelial survival, forms endothelialized channels, induces angiogenic sprouting, or generates perfusable and functionally mature vascular structures. Therefore, design elements should be linked to vascular architecture, matrix mechanics, perfusion strategy, cellular organization, molecular transport, and the intended endpoints of angiogenic function 8, 15.

For clarity, this section first separates structural device materials from ECM/hydrogel materials and then links material choice to physical cues, biological inputs, validation readouts, and reporting standards.

### 4.1 Materials Selection

Material selection directly affects the manufacturability, biological interpretability, and reproducibility of AoC readouts. In this review, materials are divided into two functional categories. Structural device substrates, including PDMS, cyclic olefin copolymer (COC), cyclic olefin polymer (COP), polymethyl methacrylate (PMMA), polycarbonate (PC), polystyrene (PS), glass, silicon, and selected photocurable resins, mainly determine channel fabrication, optical compatibility, gas exchange, chemical resistance, bonding, surface modification, and scalability. ECM-like matrices—including collagen, fibrin, Matrigel, gelatin methacryloyl (GelMA), polyethylene glycol (PEG)-based matrices, hyaluronic acid (HA)-based matrices, and decellularized extracellular matrix (dECM)—mainly determine endothelial invasion, matrix remodeling, lumenization, sprout guidance, and vascular maturation 8, 15, 41, 75. This separation is important because a device substrate optimal for fabrication or drug compatibility may not support endothelial morphogenesis, whereas a hydrogel that promotes sprouting may not be suitable for standardized drug testing.

PDMS remains widely used for early-stage AoC development because of its optical transparency, relatively low autofluorescence, elasticity, gas permeability, and compatibility with soft lithography 8, 15, 76. These properties support rapid prototyping and geometry optimization, live imaging, pneumatic actuation, cyclic stretch, hydrogel integration, and perfusion studies. Their value is application dependent. For example, gas permeability facilitates O_2_/CO_2_ exchange but, in small-volume devices, can coexist with water-vapor loss, evaporation-mediated osmolality drift, and difficulty maintaining defined oxygen gradients; these effects require application-specific control 15, 44, 77. Native PDMS is hydrophobic, but its surface properties can be deliberately tailored. Oxygen or air plasma treatment temporarily renders the surface hydrophilic; it also enables strong bonding to glass or another PDMS layer and facilitates channel filling and subsequent functionalization 15, 76. Plasma treatment is therefore a useful fabrication and surface-engineering tool; the practical limitation is time-dependent hydrophobic recovery, which progressively reduces surface wettability. Plasma conditions, post-treatment storage, and the interval between treatment and use should therefore be controlled and reported 76. Other limitations should be considered according to the intended context of use. PDMS can swell in some organic solvents 76, and incomplete curing may allow residual uncrosslinked oligomers to leach into the culture medium 78. PDMS can alter drug exposure through surface adsorption and bulk absorption; collectively, these processes are termed sorption. Sorption may initially reduce the concentration of freely available compounds and later permit the release of retained compounds, thereby changing the magnitude, spatial distribution, and temporal profile of cellular exposure 79-80. The extent of sorption depends on the physicochemical properties of the compound, the geometric structure of the device, the surface area and volume ratio, the exposure time, the flow conditions and the composition of the culture medium 79-81. Surface modification should be selected according to the expected function: ECM coating is used to support endothelial adhesion, PEG treatment can reduce nonspecific fouling, and sol-gel or parylene coating can reduce the interaction between materials and compounds 8, 76. No single treatment can eliminate all PDMS-related effects. AoC research based on PDMS should report the base-to-curing-agent ratio, curing and post-curing conditions, bonding method and plasma treatment conditions, and should also report the interval between plasma treatment and use, sterilization method, coating and storage conditions, and measures for controlling evaporation and oxygen levels. When drug exposure is the experimental endpoint, the compound-recovery or mass-balance measurements should also be reported 79-80.

Thermoplastics and other rigid substrates are practical alternatives in drug testing and the scalable manufacture of reproducible devices. Each material needs to be evaluated separately. COC, COP, PMMA, PC and PS can be processed by injection molding, hot embossing or micromilling. Some photocurable resins can be used for additive manufacturing 78. Studies have shown that the adsorption of some material-compound combinations is lower than that of PDMS, but this result should not be applied to all rigid polymers 78, 80. COC and COP have optical transparency and low water uptake, and their compound recovery still depends on molecular properties, surface treatment and exposure conditions 79, 82. Compared with PDMS, thermoplastics usually have lower gas permeability and greater stiffness. When used, specific steps may need to be taken for ECM coating before bonding, joint integration, surface activation or endothelial seeding. Glass provides optical transparency, low autofluorescence and chemical stability. Silicon supports precision micromachining and sensor integration, but it is not transparent to visible light, so a transparent window is required for conventional optical microscopy 15, 83.

Hydrogels should be considered separately from the substrate of the structural device. They create a three-dimensional ECM-like microenvironment and regulate endothelial invasion, polarization, sprouting, lumen formation, anastomosis, mural-cell recruitment and vascular stability 40, 84. Natural hydrogels such as type I collagen and fibrin provide integrin binding sites and can be remodeled through proteolysis, thus supporting endothelial migration and sprouting 85-86. Collagen I is widely used to study the invasion of existing endothelial structures; fibrin is often used to support cell-assisted network assembly. Matrix concentration, polymerization conditions, pH, temperature, and degradation rate should be controlled because they jointly affect stiffness, ligand density, permeability, and pore architecture 85-86. Matrigel provides basement-membrane components and proangiogenic cues, but its murine tumor origin, ill-defined composition, and lot-to-lot variability hinder standardization and clinical translation 87-88. Engineering and semisynthetic matrices include GelMA, PEG-based hydrogels, hyaluronic-acid derivatives, and engineered protein matrices. Their chemical composition determines the tunable range of stiffness, ligand presentation, protease-mediated degradation and microstructure 30, 84, 86, 89. Some formulations require the addition of adhesive ligands or protease-cleavable cross-linkers. In the photocrosslinking systems, the toxicity, light dose and spatially nonuniform crosslinking of the initiator may affect the performance. Human cell-derived matrices and dECM hydrogels retain aspects of tissue biochemical complexity, but their source variation, processing methods, and batch composition need to be characterized; under applicable circumstances, decellularization efficiency and residual DNA content should also be characterized 87, 90. Matrix architecture is another important consideration. Bulk hydrogels provide space for endothelial invasion, whereas patterned interfaces and channels guide tissue organization and connect engineered vessels to perfusion pathways 84, 91. AoC research should report the material source and relevant batch information, as well as gelation or cross-linking conditions, measured mechanical properties and degradability; if available, it should also report the validation results of porosity or permeability, interface geometry, and lumenization and perfusion.

Material selection should follow the intended function of the AoC platform. PDMS remains useful for exploratory work requiring live imaging, dynamic deformation, or rapid design iteration. Thermoplastic and glass-based platforms may offer advantages for standardized pharmacological assays and scalable fabrication, provided their material–compound interactions are validated. Natural hydrogels support endothelial sprouting and self-assembly, whereas engineered hydrogels can provide more independent control of stiffness, degradability, and ligand presentation. Hybrid systems can combine complementary properties. A suitable platform should preserve endothelial viability and the vascular process of interest while supporting the required measurements of morphology, perfusion, permeability, mural-cell coverage, mechanical responsiveness, molecular transport, and drug response. Minimum reporting should cover substrate identity, ECM formulation, surface modification, bonding and sterilization, measured mechanical properties, channel geometry, relevant compound-sorption controls, fabrication method, and assay-specific validation criteria.

### 4.2 Physical Factors

In AoC systems, physical factors define the mechanical and transport context in which endothelial cells invade the matrix, form lumens, stabilize junctions, and become perfusable. These variables should be interpreted according to the specific model architecture. In sprouting AoC systems, the key question is how a pre-existing endothelialized vessel responds to luminal flow, interstitial flow, pressure gradients, and ECM mechanics. In vasculogenesis-like self-assembled microvascular networks, the same parameters mainly regulate *de novo* network assembly, anastomosis, perfusion, and regression 14, 40. Therefore, physical factors should be reported as quantitative design inputs rather than as generic descriptions such as “dynamic culture” or “perfusion”.

Intraluminal flow and WSS are the most commonly manipulated mechanical variables but should be distinguished: flow mediates advective solute transport, whereas WSS is the tangential traction exerted on the endothelium. Reported WSS values in mature human vessels are approximately 0.1–0.5 Pa in veins, 0.3–1.3 Pa in arteries, 0.5–2.2 Pa in the aorta, and 1–6 Pa in arterioles; reported capillary estimates span approximately 0.3–9.6 Pa but vary markedly with vessel diameter, intermittent perfusion, and measurement method 92. These values are vessel-bed references rather than universal chip targets. WSS should be estimated from channel geometry, local vessel diameter, flow rate, and medium viscosity, or determined using validated flow-field measurements and computational modeling. Where possible, local flow fields should be measured using particle tracking velocimetry (PTV) or micro-particle image velocimetry (μPIV), and local WSS should be estimated using validated analytical expressions or CFD. An ideal straight circular lumen can provide a more symmetric shear-stress field 9, 92. The biological effect of shear is stage- and context-dependent. Laminar shear can support endothelial alignment, junctional organization, and barrier maturation 92, whereas disturbed flow can promote inflammatory and matrix-remodeling responses 8, 92; neither response alone demonstrates angiogenesis. In a perfused parent-vessel model, shear alone did not initiate sprouting: a proangiogenic gradient was required, and 0.15 or 0.5 Pa reduced endothelial invasion, with 0.5 Pa shifting invasion from parent-connected multicellular strands toward scattered cells 46. In one self-assembled vascular network model, remodeling without detectable damage was associated with WSS of 0.01–0.04 Pa, whereas rupture was observed in regions above approximately 0.04–0.06 Pa 93. The response to fluid flow also varies with the level of VEGF: under low VEGF conditions, flow promotes vascular pruning and network refinement; under high VEGF conditions, it leads to excessive growth, and bevacizumab can restore flow-induced remodeling 93. Therefore, the appropriate WSS range depends on the vascular structure, maturity stage and biochemical environment.

Interstitial flow is distinct from intraluminal perfusion and should be treated as an independent design variable. In tissues, it arises from pressure gradients associated with processes such as transvascular filtration and lymphatic drainage. Reported interstitial velocities in developing tissues are approximately 0.1–10 μm/s, coupling solute transport with mechanical cues 13. Its effects depend on matrix transport properties. In one comparison, pressure-driven convection disrupted gradients in microporous collagen, whereas a nanoporous synthetic matrix restricted interstitial flow and maintained the imposed gradient 46. In a self-assembled microvascular network model, pressure differences of 10–30 mm H₂O (approximately 98–294 Pa) increased vascular density, diameter, and perfusability; the resulting velocity depended on the pressure difference and matrix permeability 53. Studies should therefore report the pressure difference, measured or calculated Darcy velocity, flow direction relative to sprouts, matrix permeability, and temporal profile 13, 53.

Hydrostatic pressure and cyclic stretch provide additional mechanical inputs but should not be generalized to every AoC system. In one study using 3D organotypic endothelial cultures and aortic rings, continuous hydrostatic pressure of 20 mmHg increased sprout area, whereas 55 mmHg was less effective and produced defective sprouting 94. These experimental conditions were selected to approximate particular microvascular pressure environments and should not be treated as universal *in vivo* pressure values. Cyclic stretching is most relevant for mechanically deformable vascular beds, including large or muscular vessels, pulmonary and cardiac vascular beds, and aneurysmal or atheroprone environments 7-8, 92. When cyclic stretch is applied, amplitude, frequency, waveform, duty cycle, substrate stiffness, and the phase relationship with flow should be reported. In human saphenous vein endothelial cells, 12.5-15% multidirectional stretch at 1 Hz increased oxidative stress and inflammatory activation and induced actin remodeling relative to 2.5-5% stretch; however, this monolayer result does not directly establish an effect on angiogenesis 95. For capillary sprouting AoC, stretch should be considered model-specific rather than mandatory.

Matrix mechanical properties determine how forces are transmitted to endothelial cells and how easily cells invade, remodel, and lumenize the ECM. Stiffness, viscoelasticity, degradability, pore size, ligand density, and fiber architecture should be considered together rather than reduced to hydrogel concentration 14, 40. In a tunable synthetic matrix model, long, perfusable tubes formed when matrix adhesiveness supported initial collective endothelial invasion and sufficient proteolytic degradability permitted subsequent matrix remodeling and lumen opening 30. Resistance to proteolytic cleavage can also switch the migration mode of angiogenic sprouts, underscoring the inseparability of matrix mechanics and degradability 96. Because physiological matrix mechanics are tissue- and method-dependent, no single modulus is universally appropriate; the selected range should be justified for the target tissue and measured by rheology, indentation, or a comparable method under the same gelation and culture conditions used in the chip 14, 40.

Physical design also determines whether a vascular structure is morphologically vascularized or functionally competent 30, 97. Vessel diameter, cross-sectional shape, branching hierarchy, multilayer organization, and mural-cell support should be matched to the biological question. Capillary-like networks are typically evaluated by small lumen diameter, high branching density, perfusability, barrier integrity, and pericyte coverage, whereas arteriole- or venule-like models should additionally consider larger channel diameter, circumferential mural-cell organization, pressure responsiveness, and flow-dependent endothelial alignment 58, 98-99. A straight endothelialized channel is primarily suited to controlled studies of luminal flow and endothelial shear responses 92. Circular channels with circumferentially organized vascular smooth muscle cells can additionally reproduce vascular-wall organization and flow-induced endothelial alignment 98. A pre-patterned parent-vessel channel models sprouting angiogenesis when endothelial sprouts can be traced to that vessel as they invade the adjacent 3D matrix. Subsequent lumen formation and perfusion establish additional structural and functional endpoints 30. Conversely, self-assembled networks can model perfusable microvascular beds and vascularization but may represent vasculogenesis-like assembly unless sprouting from a defined parent vessel is explicitly demonstrated 14, 40. Functional validation should include lumenization, anastomosis, dextran or bead perfusion, permeability, barrier-protein continuity, mural-cell recruitment, regression over time, and response to pro- or antiangiogenic perturbations 30, 97.

Reporting physical factors is essential for translational interpretation. In drug testing, flow affects exposure, residence time, local concentration, endothelial state, and transport across the vascular wall. Sorption to chip materials, including PDMS and other hydrophobic polymers, can further reduce the effective concentration of lipophilic compounds 79, 81. For therapeutic proteins or growth factors engineered with ECM-binding domains, matrix affinity can prolong local retention and alter spatial exposure 100. In serum-containing media, protein binding reduces the unbound fraction of many small-molecule drugs and can affect apparent activity 101. Systemic pharmacokinetic prediction generally requires additional organ compartments and integration with physiologically based pharmacokinetic (PBPK) or related computational models; a digital twin coupled to a three-organ microphysiological system provides one example 102.

Physical stimuli do not uniformly promote angiogenesis. Their effects reflect coupled mechanics and transport and depend on magnitude, direction, duration, vascular architecture, maturity, matrix properties, and biochemical context. Physiological ranges should therefore inform model-specific boundary conditions and be linked to process-specific endpoints, such as sprout initiation and extension, lumenization, perfusion, pruning, permeability, rupture, or regression.

### 4.3 Biological Factors

The biological design of the AoC platform determines its vascular phenotype, tissue specificity, disease relevance and translational potential 103. Among these factors, the origin of endothelial cells and the composition of supporting cells are two core choices. The identity of endothelial cells affects their proliferative capacity, barrier properties, inflammatory responses, and angiogenic behavior 104-109. Human umbilical vein endothelial cells (HUVECs) are easy to obtain and widely used. They are practical cell sources for sprouting experiments and self-assembly networks, but they cannot generally replace organ-specific endothelial cells. Endothelial colony-forming cells (ECFCs) and primary microvascular endothelial cells provide other options, and their applicability depends on the target tissue and experimental purpose; donor differences, passage number, tissue source and phenotypic drift caused by culture need to be taken into account 104-105. Brain microvascular, glomerular, dermal, and hepatic sinusoidal endothelial cells can provide organ-related phenotypes; arterial endothelial cells can be used to study vessel-type-specific biology 106, 109. Human pluripotent stem cell (hPSC)-derived endothelial cells, including induced pluripotent stem cell (iPSC)-derived endothelial cells, provide renewable and potentially patient-matched sources for genetic disease modeling and personalized testing. However, before these cells are used for quantitative angiogenesis studies or drug screening, their differentiation efficiency, maturity, endothelial phenotype and batch-to-batch consistency must be verified 105-106.

Endothelial cells alone generally do not form long-term stable, perfused, and physiologically meaningful vessels. Pericytes and vascular smooth muscle cells promote mural-cell coverage and basement-membrane deposition and contribute to barrier stability and contractile function 35, 107. Fibroblasts and mesenchymal stromal cells are mainly responsible for ECM remodeling, while providing paracrine angiogenic cues and shaping the local matrix microenvironment 108, 110. The PDGF-BB/PDGFRβ axis is a key regulator of pericyte recruitment and vascular stability 35, 111; Ang-1/Tie2 signaling helps maintain endothelial quiescence and junctional stability 36. Recent microfluidic studies show that there may be significant differences in vascularization efficiency among pericytes, primary stromal cells and iPSC-derived mesenchymal stromal cells; therefore, these cell types should not be regarded as interchangeable 108, 110. Biochemical cues should be introduced as controlled signals rather than as generic additives. Vascular endothelial growth factor A (VEGF-A)/VEGFR2 signaling promotes endothelial migration and tip-cell competence 2. DLL4-Notch lateral inhibition restricts excessive tip-cell selection and supports stalk-cell specification 34, 112. Fibroblast growth factor 2 (FGF-2) supports endothelial proliferation and survival 113.

In AoC platforms, angiogenic factors can be delivered as stable gradients, temporally staged signals, or compartmentalized paracrine cues released by stromal cells, mural cells, immune cells, or organ-specific cells 104, 112, 114. This design logic links single-factor angiogenesis assays to tissue-vascular interaction models. For example, co-culture induction systems have shown that vascular and neuronal network formation can be dynamically coupled on chip, indicating that angiogenic cues may also arise as endogenous tissue-derived signals during organ-level vascularization 115. Disease-associated mediators, including inflammatory cytokines, tumor-derived angiogenic factors, hypoxia-related signals, and fibrotic mediators, should be introduced only when their concentration, timing, and cellular source match the intended disease context.

Immune and lymphatic components should be incorporated when the goal is to model inflammatory angiogenesis, tumor angiogenesis, or vascular-lymphatic crosstalk. Macrophage effects on angiogenesis are activation-state- and microenvironment-dependent 116. Accordingly, studies should report macrophage source, conditioning or polarization method, phenotype markers, and spatial placement. Lymphatic endothelial components should not be conflated with blood endothelial networks. Lymphatic vessel-on-chip models can be used to investigate lymphatic sprouting, discontinuous endothelial-junction formation, and the independent or combined effects of interstitial flow and VEGF-A/VEGF-C signaling on lymphangiogenic remodeling 117. Accordingly, immune or lymphatic modules increase physiological relevance only when their contributions to angiogenic or lymphangiogenic processes are evaluated using corresponding functional readouts; otherwise, additional cellular complexity may reduce mechanistic clarity and assay robustness.

Biological relevance should be judged by readouts that match the intended biological question. Structural metrics, including sprout length, sprout number, branch density, network area, vessel diameter, and anastomosis frequency, characterize vascular morphogenesis but do not by themselves establish vascular function 49. Functional validation should therefore combine direct assessment of perfusion and vascular permeability with context-specific therapeutic-response endpoints 50, 118. Molecular readouts should be process-specific: VE-cadherin, ZO-1, and claudin-5 for junction maturation and permeability-related phenotypes 107, 119; VEGFR2, DLL4, and the Notch target genes HES1 (hes family bHLH transcription factor 1) and HEY1 (hes-related family bHLH transcription factor with YRPW motif 1) for assessing VEGF–DLL4–Notch-associated endothelial states 9, 34; PDGFRβ, neuron-glial antigen 2 (NG2), and α-smooth muscle actin (α-SMA) for assessing mural-cell identity and coverage, with marker combinations and spatial localization interpreted together because none is universally pericyte-specific 14, 108; and vascular cell adhesion molecule 1 (VCAM-1), intercellular adhesion molecule 1 (ICAM-1), cytokine release, and leukocyte adhesion or transmigration for inflammatory endothelial activation 4, 120-121. Oxidative-stress markers should be included only when oxidative injury is explicitly part of the intended disease model. Integrated sensors can additionally support continuous monitoring of oxygen conditions and metabolic activity during on-chip culture 122. Image-based and model-informed analysis can further strengthen AoC validation. Deep learning-assisted AoC studies have used endothelial nuclear segmentation, vessel skeletonization, and 3D image-processing pipelines to quantify vessel length, width, number, branching, and nuclear distribution 123. Angio-Net further showed that bright-field images can be converted into virtual fluorescence images for label-free extraction of vascular morphometric features 124. Machine-learning-based vascular network quality index analysis can also link vascular architecture with oxygen-transport capacity, providing a more functional estimate of vascular quality than isolated structural descriptors 125. Current and potential artificial intelligence (AI) applications include endothelial network segmentation, sprout-tip tracking, branch-point and endpoint quantification, lumen and perfusion detection, leakage-area analysis, topology-based maturity assessment, batch-quality control, phenotype clustering, and integration with CFD, oxygen-transport modeling, or single-cell/transcriptomic readouts 9, 123-126. However, small training datasets, limited shared benchmark images, inconsistent metadata, heterogeneous endpoints, manual annotation bias, and weak cross-platform validation may limit model generalizability across different chip geometries, cell sources, matrices, and imaging systems 127.

For reproducibility, AoC studies should report endothelial cell source, donor information, passage number, differentiation protocol if stem-cell-derived cells are used, endothelial subtype markers, supporting-cell type and ratio, immune or lymphatic cell activation state, ECM composition, growth-factor concentrations, gradient-generation method, culture timeline, perfusion status, imaging modality, image-analysis workflow, segmentation or quantification method, software or model version, AI training and validation datasets when applicable, and statistical treatment of network heterogeneity 49, 104-105, 122. In this way, biological factors should define which angiogenic mechanism, disease process, or therapeutic response is being tested rather than simply increasing model complexity.

To synthesize the design principles discussed above, Table 3 summarizes the major AoC design domains, including material and matrix selection, physical cues, biological modules, and analytical validation. Rather than ranking materials or platforms, the table links representative implementations to controlled variables, suitable applications, validation readouts, and reporting requirements.

## 5. Applications of AoC Systems

### 5.1 Modeling Physiological Development and Vascular Pathologies

AoC models have evolved from simple endothelialized perfusion devices into controllable microphysiological platforms for investigating tissue-specific vascular development, repair, and disease. Their major advantage is the ability to examine how endothelial cells interact with stromal or parenchymal cells, the extracellular matrix, soluble signals, and hemodynamic cues within a defined microenvironment. The criteria used to evaluate these models should therefore be matched to the biological process being studied—for example, perfusion and anastomosis in vascularized organoids, barrier integrity in ocular or neurovascular models, inflammatory endothelial activation in atherosclerosis, and abnormal permeability or drug delivery in tumor vasculature. The following examples illustrate how AoC platforms have been applied to physiological tissue reconstruction and vascular disease modeling.

In physiological and developmental contexts, endothelial cells are not merely passive conduits but instructive niche components that regulate organ development and tissue maturation. Limited internal convective transport and endothelium-derived angiocrine signals can contribute to the immaturity of avascular organoids. Kroll *et al*. established a perfusable vascularized kidney organoid-on-chip by positioning kidney organoids and a HUVEC-lined macrovessel in adjacent addressable channels embedded within an ECM compartment 128 (Figure 4A-i). Organoid-derived microvessels extended through the ECM and formed lumen-to-lumen anastomoses with the macrovessel, enabling fluorescent dextran and red blood cells to enter the organoid microvasculature. Together with related kidney organoid-vasculature interaction platforms 129, this model provides a controlled framework for investigating organoid vascularization and endothelial-epithelial crosstalk and may support future nephrotoxicity testing. However, glomerular filtration-barrier function, segment-specific endothelial identity, and long-term vascular stability require further validation before these systems can be considered functionally mature kidney models.

AoC systems are also useful for reconstructing specialized vascular barriers and tissue-repair microenvironments. In ocular angiogenesis models, injection-molded microfluidic platforms can reconstruct angiogenic sprouting within geometrically defined vascular networks, whereas retinal pigment epithelial-endothelial co-culture chips can be used to examine choroidal angiogenesis-related epithelial-endothelial crosstalk under altered metabolic or hypoxic conditions 130-131 (Figure 4A-ii). Such systems are particularly relevant for diseases such as age-related macular degeneration, because they permit controlled analysis of epithelial-endothelial crosstalk and antiangiogenic responses in a compartmentalized microenvironment 130-131. However, the interpretation of these platforms should distinguish between morphological sprouting and true barrier dysfunction, which requires complementary assessment of permeability, tight-junction organization, and polarized epithelial transport 131. Similarly, a vascularized 3D skin-on-chip combined a stratified epidermis with a fibroblast-containing dermis and a perfusable endothelialized microvascular network that maintained barrier function and generated endothelial sprouts in response to angiogenic stimulation 132 (Figure 4A-iii). Their main strength is the integration of perfusion with stratified tissue architecture, whereas their limitations include incomplete immune representation, simplified lymphatic drainage, and the need to verify whether endothelialized channels undergo angiogenic remodeling or mainly serve as perfusion conduits.

Once these physiological baselines are established, AoC models can also be used to examine how vascular function shifts under pathological conditions. In cardiovascular disease models, hemodynamic control is essential because disturbed or low shear stress contributes to endothelial dysfunction, leukocyte adhesion, smooth muscle cell migration, and plaque initiation. A recent 3D atherosclerosis chip model integrated human aortic smooth muscle cells, endothelialized perfusable vascular channels, THP-1 macrophage-derived foam cells, and circulating monocytes within a 3D ECM environment, recapitulating key features of early atherosclerotic lesions, including subendothelial foam cell accumulation and monocyte recruitment 121 (Figure 4B-i). This type of model is more informative than static culture for studying flow-dependent inflammatory activation, but it mainly represents early plaque-associated vascular remodeling and should not be overinterpreted as a complete model of advanced atherosclerosis, calcification, plaque rupture, or systemic lipid metabolism 121. AoC systems are also valuable for modeling microvascular regression and defective angiogenesis in fibrotic diseases. In systemic sclerosis, the vascular phenotype is characterized by impaired angiogenic repair, endothelial dysfunction, and progressive microvascular rarefaction. High-throughput microvessel-on-chip models have been used to quantify defective angiogenesis and demonstrate prevention of cytokine-induced microvascular destabilization by inhibiting transforming growth factor β (TGF-β)/activin receptor-like kinase 5 (ALK5) signaling 133 (Figure 4B-ii). These platforms provide useful readouts for antifibrotic and proangiogenic screening, but their translational relevance depends on whether microvessel morphology is combined with functional endpoints such as perfusion, leakage, mural-cell association, and patient-derived cell responses.

Tumor angiogenesis is another major application because tumor cells, stromal components, hypoxia, inflammatory mediators, and matrix remodeling jointly generate immature, tortuous, and leaky neovasculature. A vascularized tumor-on-chip model incorporating metastatic renal cell carcinoma spheroids and ECs reproduced directional tumor-induced sprouting and vascular disruption after bevacizumab treatment 134 (Figure 4B-iii). The study compared control ECs with ECs engineered to overexpress prostate-specific membrane antigen (PSMA), and also detected tumor-associated induction of PSMA in control ECs. It enabled real-time confocal imaging, perfusate-based molecular analysis, intravascular drug delivery, and comparison with an *in vivo* tumor model 134.

Perfusable vascularized tumor-spheroid chips have enabled longitudinal analysis of chimeric antigen receptor T (CAR-T) cell transport through vessels, adhesion, extravasation, tumor-cell killing, and cytokine responses 135. Separate tumor-on-chip models have been used to quantify natural killer cell migration, cytotoxicity, and microenvironment-induced exhaustion 136, whereas skin-on-chip systems have enabled controlled analysis of T-cell transendothelial and transepithelial migration 137. Broader vascularized skin models with perifollicular vascular plexuses have also been used to investigate cutaneous immune-cell trafficking 138. These endpoints characterize immune-cell trafficking and therapeutic function rather than angiogenesis itself and should therefore not be interpreted as evidence that a platform reproduces angiogenic vessel formation. Despite these capabilities, current tumor AoC models incompletely reproduce immune and stromal heterogeneity, systemic pharmacokinetics, and temporal tumor evolution, and they may not clearly distinguish antiangiogenic effects from cytotoxicity, anti-migratory activity, or barrier-disrupting responses.

Across these examples, the core value of AoC lies in its ability to reconstruct disease-relevant vascular functions under defined experimental control. Therefore, AoC disease models should not be judged solely by the presence of endothelial networks but must explicitly define the specific vascular process being represented: organoid vascularization, angiogenic sprouting, endothelial barrier formation, vascular regression, inflammatory activation, tumor neovascularization, or vascular maturation. Future disease models should also incorporate immune–vascular and blood vascular–lymphatic interactions, especially when these processes play a central role in disease pathogenesis. For example, lymphatic vessel-on-chip systems have demonstrated that interstitial flow and VEGF-A/VEGF-C signaling regulate lymphatic sprouting and junctional organization, while lymphatic networks integrated into lymph node-on-chip models can enhance immune cell maintenance and chemokine secretion 117, 139. Immune-cell drainage and tumor-cell entry into lymphatic vessels, however, require dedicated trafficking or tumor–lymphatic models and cannot be inferred from lymphatic sprouting or endothelial-junction assays alone. Thus, coupling blood vascular, lymphatic, immune, and stromal modules is particularly valuable when the biological question concerns edema, immune-cell trafficking, tumor dissemination, chronic inflammation, or therapeutic delivery. In all cases, cell sources, matrix composition, flow conditions, structural endpoints, and functional validation should be aligned with a clearly defined biological or therapeutic question.

### 5.2 Drug Screening and Toxicity Evaluation

Microfluidic angiogenesis assays have demonstrated that VEGF-driven endothelial cell migration and sprouting can be quantitatively characterized under well-defined biochemical gradients, enabling the evaluation of antiangiogenic drugs 140. Human microvessel-based sprouting assays further showed that antiangiogenic responses can be measured in parallel with endothelial barrier readouts, thereby distinguishing sorafenib-induced barrier disruption from the comparatively junction-preserving effect of sunitinib 118. These studies establish a key advantage of AoC drug-screening models: efficacy should not be inferred solely from reduced sprout length, but should be interpreted in combination with perfusion, barrier integrity, and vascular morphology. Recent high-throughput OoC screening has extended this logic from small candidate compound libraries to phenotypic drug discovery. In a screen encompassing 1,537 kinase inhibitors, reduced sprouting was used as an efficacy endpoint, whereas loss of parent microvessel integrity served as a toxicity endpoint, thus effectively distinguishing angiogenic inhibition from broad vascular injury 49.

Perfusion is particularly important for drug evaluation because it defines the exposure route, exposure duration, residence time, and spatial distribution of compounds. Coaxially bioprinted vessel-on-chip systems can maintain lumenized HUVEC vessels under continuous perfusion and enable real-time monitoring of concentration-dependent bevacizumab-mediated sprouting suppression 57. Perfused 3D angiogenic sprouting platforms and standardized high-throughput systems such as Anchor-IMPACT show that hydrogel patterning, endothelialized channels, and scalable image-based quantification can improve the reproducibility of antiangiogenic screening 48, 97. Scalable assays using perfused human iPSC-derived endothelium further broaden the testing space by incorporating genotype- or patient-relevant endothelial responses while preserving key angiogenic features, including tip-cell formation, directional sprouting, and lumen formation 141. Therefore, AoC drug-screening studies should specify the delivery route, flow-driving method, flow rate, estimated WSS or interstitial flow velocity, medium viscosity, exposure duration, dosing schedule, and whether concentration gradients were verified by tracer imaging or computational transport modeling. For toxicity evaluation, reduced sprouting should be interpreted as selective antiangiogenic activity only when endothelial viability, parent-vessel integrity, junctional continuity, perfusion, and leakage are assessed in parallel 49, 118. This multiparametric strategy helps distinguish selective inhibition of angiogenic sprouting from direct endothelial cytotoxicity, barrier-disruptive vascular injury, and nonspecific vascular collapse.

Vascularized tumor-on-chip systems extend AoC-based drug evaluation from isolated endothelial sprouting assays toward preclinical evaluation of vascular-targeted therapies, analysis of vascular delivery barriers, and imaging-compatible vascular phenotyping. A vascularized metastatic renal cell carcinoma-on-chip model enabled real-time, high-resolution observation of tumor-induced endothelial sprouting and was used to evaluate bevacizumab-mediated disruption of tumor-vessel interactions 134. The same study compared on-chip bevacizumab responses with an *in vivo* tumor model, strengthening the translational interpretation of the chip-based vascular readouts. Another vascularized tumor-on-chip system fused tumor spheroids with HUVEC spheroids to model tumor-angiogenesis interplay and compare tumor-type-specific angiogenic phenotypes and drug responses 142. These examples indicate that AoC platforms can support not only antiangiogenic efficacy testing, but also vascular delivery analysis, tumor-endothelium crosstalk studies, and image-based evaluation of vascular-targeted treatment responses 134, 142.

Despite these advantages, AoC-based drug screening still has several important limitations. PDMS devices can sorb hydrophobic small molecules through surface adsorption and bulk absorption, causing the concentration reaching cells to deviate from the nominal medium concentration; therefore, researchers have developed simulation-based and experimental approaches to estimate the spatial and temporal concentration profiles of drugs in PDMS organ chips 80. In hydrogel-containing vascular chips, the effective drug exposure may also be influenced by matrix diffusion distance, matrix porosity, medium composition, and local residence time, so concentration gradients and recovery should be further validated experimentally or computationally rather than assumed 57, 80. Single-organ AoC systems generally cannot recapitulate whole-body pharmacokinetics, hepatic metabolism, renal clearance, the full complexity of plasma protein binding, or immune-mediated toxicity without appropriate medium design, pharmacokinetic modeling, immune components, or multi-organ integration 143-144. Multi-organ chip platforms can help analyze inter-organ interactions during cancer therapy, whereas broader microphysiological-system studies emphasize the need to incorporate systemic toxicity mechanisms when evaluating new therapeutic regimens 144-145. To enhance the reliability of translational research, future AoC drug studies should include material-compatibility testing, compound recovery or mass-balance measurements, use of low-sorption thermoplastic or coated devices where appropriate, tracer-validated transport profiling, pharmacokinetic modeling, and orthogonal toxicity controls 10, 80, 143-145. If AoC systems are to move from specialized experimental platforms toward routine drug development and regulatory-facing applications, standardized device design, transparent data reporting, context-of-use definition, validation strategy, and deployment workflow remain essential.

### 5.3 Mechanistic Exploration

AoC has become an important research tool, transforming angiogenesis from a morphology-based endpoint assay into a controllable, perturbable, and imageable microphysiological process. Compared with conventional tube-formation or bead-based assays, sprouting AoC enables the simultaneous presentation of the parent vessel, ECM compartment, stromal or mural cells, soluble concentration gradients, and well-defined luminal or interstitial flow within the same field of view. This configuration allows candidate mechanisms to be tested through single-variable modulation while retaining structural and functional endpoints such as sprout initiation, tip-stalk distribution, lumen continuity, perfusability, permeability, mural-cell coverage, and long-term network remodeling. To avoid repetition of the engineering strategies discussed above, this section focuses on how AoC platforms have been used to infer causal mechanisms rather than on how the devices are fabricated.

At single-cell resolution, AoC models are beginning to reveal how endothelial heterogeneity is organized along angiogenic sprouts. Lee *et al*. combined a high-throughput 3D AoC model with single-cell RNA sequencing and showed spatially differential autophagy activation in sprouting endothelial cells, with higher activation in proliferating endothelial cells at sprout bases and stalk-like endothelial cells near sprout endpoints 39 (Figure 5A). Importantly, the reciprocal expression pattern of autophagy-related genes between stalk- and tip-like endothelial cells suggested that autophagy is not a uniform metabolic background process but may contribute to tip-stalk specification and sprout elongation in a position-dependent manner. Image-analysis pipelines further strengthen this mechanistic use of AoC by converting complex 3D vascular phenotypes into quantifiable variables. Deep learning-based angiogenesis analysis has been used to quantify sprout morphology more reproducibly than manual measurements 123, and Angio-Net further demonstrated label-free virtual fluorescence and automated morphometric analysis from bright-field images in microphysiological angiogenesis systems 124. These computational approaches are particularly valuable when they are coupled with molecular validation, because they allow changes in sprout length, branching, tortuosity, and network topology to be interpreted together with endothelial state markers rather than as isolated morphological endpoints.

AoC platforms have also clarified how non-endothelial cells regulate angiogenesis. In blood-vessel-on-a-chip systems, image-based crosstalk analysis has shown that mesenchymal stromal cells regulate sprouting angiogenesis through spatially organized endothelial-stromal interactions, which can be quantified from reconstructed 3D vessel morphology 70. Similarly, pericytes should not be regarded merely as passive stabilizing cells. Using a microfluidic angiogenesis-on-a-chip platform, Gonzalez-Rubio *et al*. demonstrated that pericytes actively enhance vascularization compared with stromal cells by promoting endothelial sprouting, vessel organization, and mural coverage through an IL-6-dependent mechanism 108 (Figure 5B). These findings highlight that pericytes function as active regulators of angiogenic remodeling rather than only structural supporters of vascular stabilization. Mechanical context modifies this mural regulation: in self-assembled vascular networks, pericytes and shear stress independently altered vascular morphology, and computational modeling linked frequent sprout formation to low-shear regions, while PDGFRβ inhibition reduced pericyte coverage 146. These studies indicate that mural cells regulate not only vessel stabilization but also network architecture, mural-cell coverage, sprout localization, and the interpretation of shear-dependent angiogenic responses 70, 108, 146.

In AoC platforms, well-defined biochemical and mechanical perturbations provide important tools for mechanistic studies. Mote *et al*. used a nanoporous hydrogel to suppress perfusion-driven interstitial convection and preserve a chemokine gradient; within the parent vessel, increasing WSS attenuated sprout invasion and altered multicellular connectivity through changes in endothelial permeability 46. Ferrari *et al*. used a microvasculature-on-chip to study ECM stiffness in a sprouting angiogenesis configuration and cyclic stretch in a separate vasculogenesis-like configuration 147 (Figure 5C). The two configurations distinguish effects on vessel-derived sprouting from effects on de novo network formation. Multilayered vascularized chips have also been used to examine fluid-dependent vasculogenesis-like network formation and subsequent network stability under different shear conditions 65. During later stages of vascular network maturation, stability depends on matrix deposition and barrier maturation; macromolecular crowding promotes cell-junction and basement-membrane formation, improves barrier properties, and thereby enhances the stability and function of three-dimensional perfusable microvascular networks 148. Collectively, these studies indicate that mechanistic angiogenesis research should report not only applied flow rates or growth factor conditions, but also key parameters such as WSS, interstitial fluid transport, matrix composition, permeability, perfusability, and barrier function.

AoC models can also resolve vascular remodeling processes that are difficult to isolate in static culture. An intussusceptive AoC model showed that endothelial delamination can generate transluminal bridges within a perfused microvessel 18. Complementary *in vivo* imaging in the zebrafish caudal vein plexus identified pillar-forming processes involving sprouting, lumen expansion, and intraluminal folding 149. These observations should not be interpreted as evidence that every pillar forms by the same mechanism. Coalescent angiogenesis describes a distinct remodeling process in which the fusion of capillary segments contributes to maturation toward a more hierarchical vascular network 150. These examples extend mechanistic analysis beyond sprout counting, while emphasizing the need to distinguish sprouting angiogenesis, intussusception, coalescent remodeling, and vasculogenesis-like self-assembly.

AoC platforms support mechanistic research by making vascular growth directly observable and experimentally tractable. Specific studies discussed earlier indicate that endothelial heterogeneity reflects spatially organized responses to local microenvironmental cues, metabolic state, and signaling context; pericytes not only stabilize vascular networks but also actively shape their organizational structure; and biomechanics act as primary regulators of vascular growth patterns rather than merely background conditions. In this sense, the greatest strength of AoC is not that it reproduces vascular complexity *in vitro*, but that it allows the key determinants of vascular assembly, remodeling, and dysfunction to be isolated and tested within the same controllable system.

## Conclusions and future perspectives

Despite rapid progress, broader use of AoC in vascular biology and translational research requires that several persistent limitations be addressed. A central challenge is to move beyond morphological resemblance toward the vascular functions required for a defined application. Endothelial sprouts, lumen-like structures, and partially perfused networks do not alone establish physiological equivalence to native microvessels. Relevant functions include regulated solute transport, selective barrier properties, dynamic remodeling, and tissue-specific endothelial activity 44. Morphologically plausible networks may still lack stable perfusion, mature junctions, appropriate mural-cell coverage, basement-membrane organization, organ-specific identity, or long-term adaptation to flow. Future work should define the minimum functional criteria needed to address a biological or therapeutic question. Tumor models may prioritize pathological permeability, tortuosity, hypoxia, and drug penetration, whereas blood-brain barrier or retinal vascular models require careful assessment of barrier selectivity, junctional integrity, and tissue-specific endothelial phenotypes. Reproducibility is a second major challenge. Device geometry, material composition, hydrogel source, matrix mechanics, cell source and passage number, co-culture ratios, perfusion, oxygen tension, and imaging workflows can all influence vascular outcomes. Interactions among these factors complicate comparisons between studies performed under different conditions. Fit-for-purpose recommendations for microphysiological systems emphasize quality management, reproducibility monitoring, and predefined performance criteria 43. AoC studies should therefore report the parameters summarized in Table 3 and establish CoU-specific acceptance criteria for perfusion, permeability, endothelial viability, mural-cell coverage, and assay reproducibility.

Another important issue is how AoC readouts should be integrated with animal models, human tissue samples, and clinically meaningful endpoints. AoC systems must not be presented as direct replacements for all *in vivo* validation, particularly when systemic pharmacokinetics, neurohumoral regulation, immune-organ crosstalk, or whole-body toxicity are central to the biological or therapeutic question under investigation. Instead, their greatest translational utility may lie in serving as an intermediate, human-relevant layer between simplified cell culture and animal or clinical studies. Recent analyses of organ-chip dissemination emphasize that successful translation requires predefined performance metrics, intended context of use, benchmarking, and clear interpretation of how chip readouts will support decision-making 10. To make this bridge credible, future studies should predefine the intended context of use and test whether on-chip endpoints such as permeability, perfusion stability, endothelial activation, vascular regression, hypoxia relief, or drug penetration show concordance with relevant animal-model data, *ex vivo* histology, imaging findings, or patient-derived data.

Biological advances will depend on reconstructing vascular interactions relevant to each CoU. Existing AoC systems reproduce endothelial sprouting and early network formation but often capture only part of the multicellular regulation of angiogenesis. Inflammatory cells influence endothelial behavior through secreted cytokines, growth factors, proteases, and reactive oxygen species; their effects depend on activation state and tissue microenvironment 116. Lymphatic components have distinct roles in AoC models, particularly in interstitial-fluid transport and immune-cell distribution. For example, a human lymph-node-on-chip model showed that a perfused lymphatic endothelial channel supported selected immune-cell populations and altered the secretory microenvironment 139. These findings do not by themselves establish macromolecular clearance, nanoparticle transport, or tumor-cell dissemination, which require dedicated functional assays. Decisions to include immune, lymphatic endothelial, stromal, or parenchymal cells should be driven by the biological question and paired with corresponding readouts, such as cytokine release, cell adhesion or transendothelial migration, vascular leakage, lymphatic drainage, tracer clearance, or drug penetration. Adding cell types without this mechanistic linkage may reduce assay robustness and obscure causal interpretation.

Computational tools and artificial intelligence can enhance quantitative AoC analysis when the task is clearly defined. They have been used for vascular-image segmentation, automated morphometry, and model-assisted estimation of oxygen transport. Potential applications also include sprout tracking, classification of perfusion or leakage patterns, batch-level quality control, and integration of time-resolved phenotypes with drug-response data. However, image-based functional predictions still require independent experimental validation. Practical limitations include small or incompletely annotated datasets, substantial variation in chip architecture and imaging protocols, and inconsistent recording of metadata and biological endpoints. Machine-learning studies should document data sources and annotation procedures, strictly separate training, validation, and test datasets, and prevent leakage between related images or samples. Performance metrics should be prespecified, uncertainty assessed, and external validation performed where feasible. Shared benchmark datasets, transparent algorithms, and experimentally validated endpoints would improve model comparison and mechanistic interpretation.

The further development of the AoC field will depend on functional validation, reproducibility, and biological specificity rather than simply increasing structural complexity. Consistent and standardized reporting of material properties, flow conditions, cell composition and vascular phenotypes will make models easier to compare and interpret. With these foundations in place, patient-specific screening, disease prediction models, and computationally guided platform optimization can be evaluated more rigorously.

## Figures and Tables

**Figure 1:**
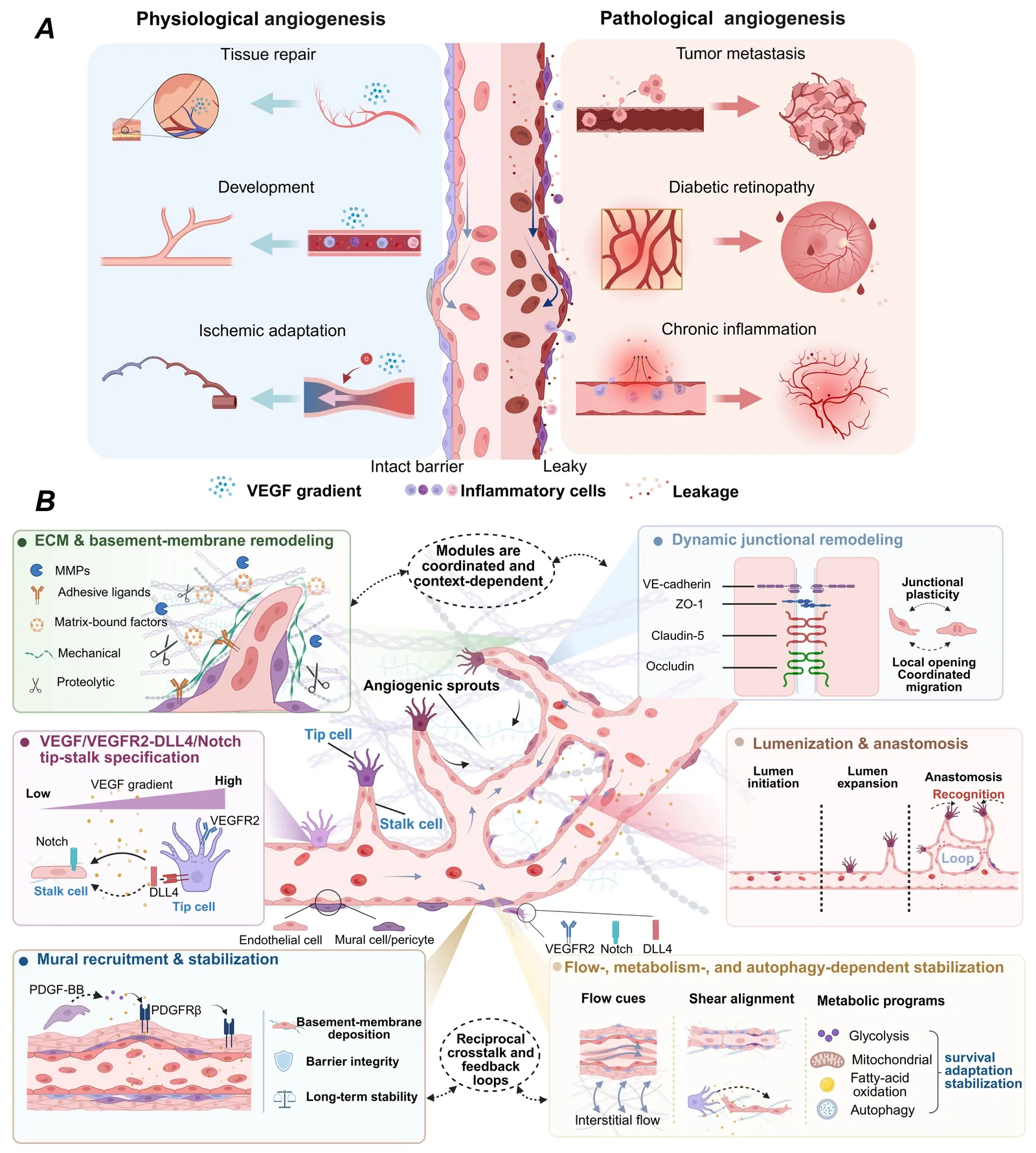
** Physiological and pathological angiogenesis and the vascular sprouting program.** (A) Under physiological conditions, an intact endothelial barrier supports tissue repair, development, and ischemic adaptation; in tumors, proliferative diabetic retinopathy, and chronic inflammation, dysregulated vascular growth can generate immature, permeable vessels and alter immune-cell trafficking. (B) Sprouting angiogenesis is controlled by overlapping and context-dependent regulatory modules, including MMP-dependent ECM and basement-membrane remodeling, dynamic remodeling of VE-cadherin-associated adherens junctions and tight junction components, VEGF/VEGFR2 and DLL4-Notch-mediated tip–stalk specification, lumenization and anastomosis, PDGF-BB/PDGFRβ-mediated mural-cell recruitment, and flow-, metabolism-, and autophagy-dependent vascular stabilization. These modules interact through reciprocal feedback rather than a strict linear sequence, providing the biological basis for functional angiogenesis-on-a-chip design and validation. Panel was created in BioRender. TAO, T. (2026) https://BioRender.com/02a4ieb.

**Figure 2:**
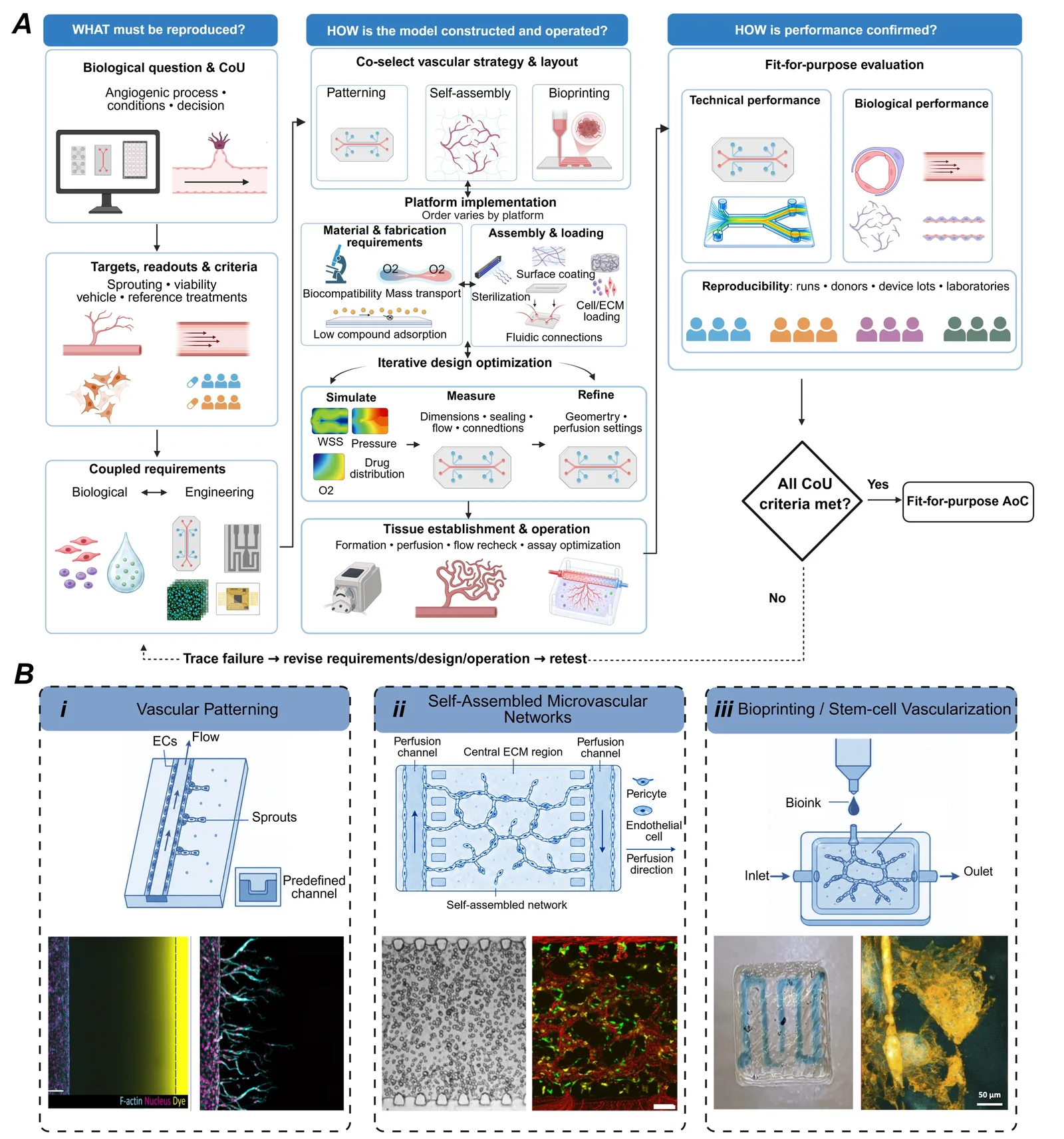
** Design-to-validation workflow and representative engineering strategies relevant to AoC.** (A) Context-of-use-driven, iterative development linking biological and engineering requirements, platform-specific construction and operation, and fit-for-purpose evaluation. (B) Representative vascular construction strategies. (i) Vascular patterning: a micromolded endothelialized tube in a nanoporous hydrogel supports chemokine-gradient-driven sprouting under controlled luminal flow and WSS. Scale bar: 100 μm. (ii) Self-assembly: ECs and pericytes form a perfusable microvascular network suitable for permeability and leukocyte-trafficking assays. Scale bar: 50 μm. (iii) 3D bioprinting: multimaterial deposition of a cell-laden GelMA matrix and a sacrificial Pluronic F127 template generates a perfusable channel within a neuroblastoma niche. Following endothelialization and flow culture, the model supports endothelial barrier formation and microvascular outgrowth toward tumor aggregates. Panel A was created by the authors in BioRender.com (TAO, T., 2026; https://BioRender.com/cq32vif). Panel B(i) was adapted from Mote *et al*. 46 under CC BY 3.0. Panel B(ii) was reproduced with permission from the Royal Society of Chemistry (Hirth *et al*. 50). Panel B(iii) was adapted from Maggiotto *et al*. 71 under CC BY 4.0. Panel B was assembled by the authors in BioRender.com (TAO, T., 2026; https://BioRender.com/an06fqy).

**Figure 3:**
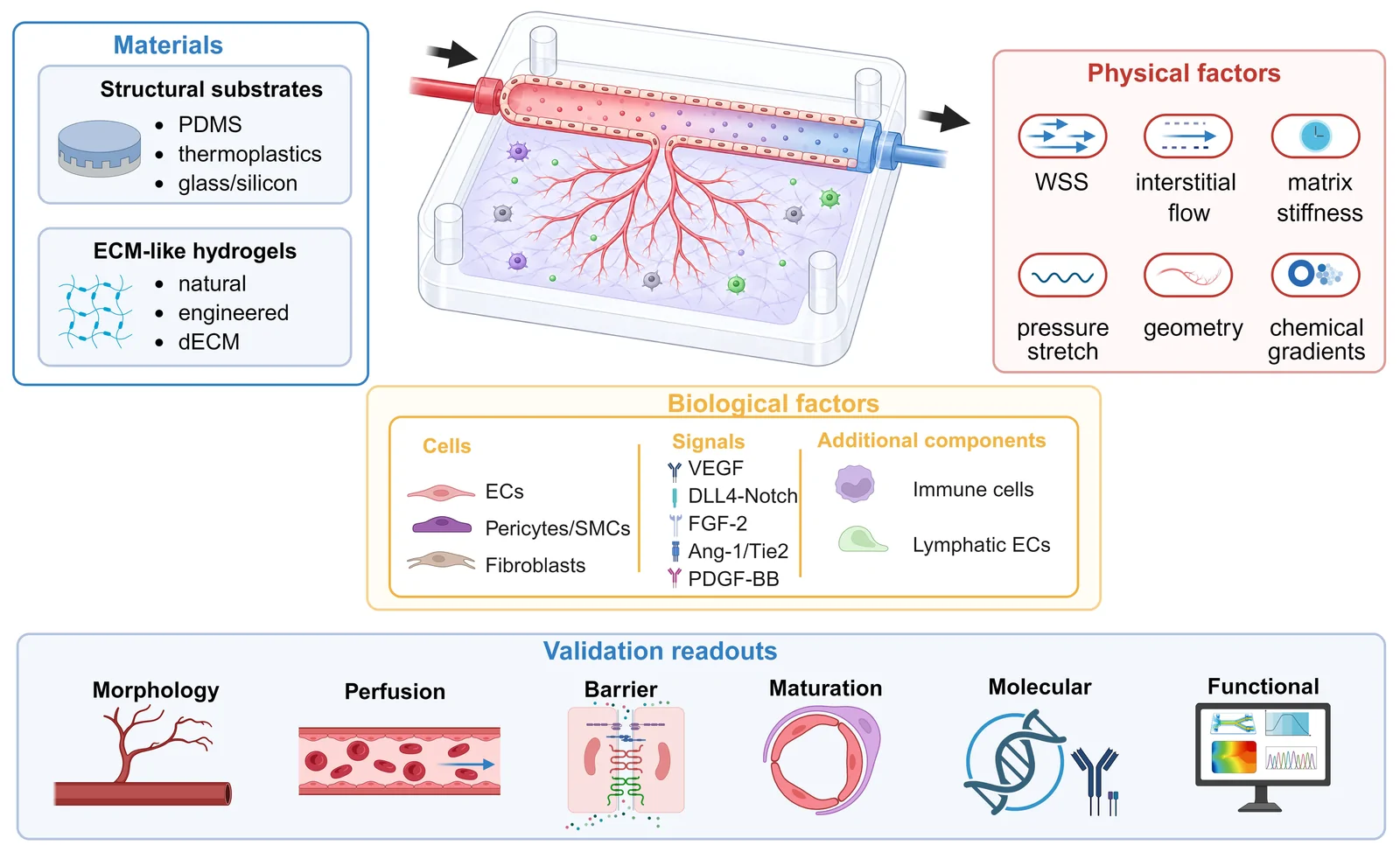
** Integrated design elements of AoC systems.** The diagram summarizes the coordinated selection of structural substrates, ECM-like matrices, physical cues, biological inputs, and validation readouts that determine AoC performance. These design elements support controlled modeling of angiogenic sprouting, vascular perfusion, barrier formation, maturation, molecular state, and disease- or drug-related responses. Panel was created in BioRender. TAO, T. (2026) https://BioRender.com/mc5as2h. **Ang-1**: angiopoietin-1; **dECM**: decellularized extracellular matrix; **EC**: endothelial cell; **ECM**: extracellular matrix; **FGF-2**: fibroblast growth factor 2; **PDGF-BB**: platelet-derived growth factor-BB; **PDMS**: polydimethylsiloxane;** SMCs**: smooth muscle cells; **VEGF**: vascular endothelial growth factor; **WSS**: wall shear stress.

**Figure 4:**
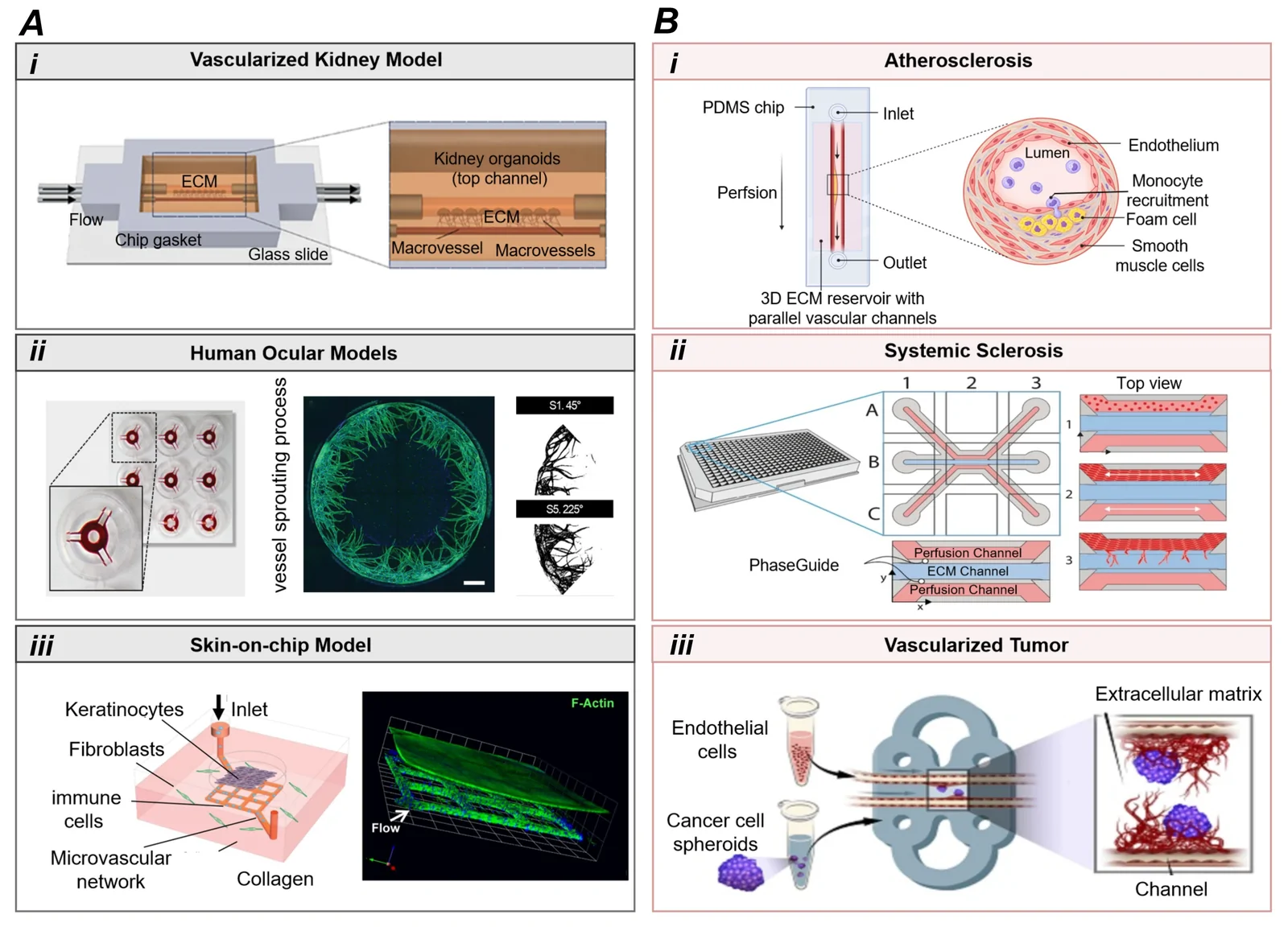
** Representative AoC platforms for organ-level reconstruction and vascular disease modeling.** (A) Physiological and tissue-repair applications. (i) Schematic of a vascularized kidney organoid-on-chip: kidney organoids and a HUVEC-lined macrovessel occupy adjacent channels in an ECM compartment; organoid-derived microvessels connect with the macrovessel to enable organoid perfusion. (ii) An injection-molded microfluidic platform supports inward or outward radial angiogenic sprouting. Fluorescence images and angular analysis illustrate spatial organization. Scale bar: 500 μm. (iii) A vascularized 3D skin-on-chip containing a stratified epidermis, a fibroblast-containing dermis, and a perfusable endothelialized microvascular network capable of angiogenic sprouting. (B) Vascular disease models. (i) A 3D atherosclerosis chip with perfusable endothelialized vascular channels embedded in an ECM reservoir and surrounded by smooth muscle cell and foam-cell compartments recapitulates early arterial lesions and monocyte recruitment. (ii) A high-throughput microvessel-on-a-chip uses phaseguides to separate the ECM and perfusion lanes; sprouts extend from an endothelialized parent vessel into the adjacent matrix. (iii) A tumor-on-chip design places tumor spheroids in an ECM compartment adjacent to perfused endothelialized channels to study tumor-induced angiogenic sprouting. Panel A(i) was reproduced with permission from IOP Publishing Ltd (Kroll *et al*. 128). Panel A(ii) was reproduced with permission from John Wiley and Sons (Ko *et al*. 130). Panels A(iii), B(ii), and B(iii) were adapted from Sun *et al*. 132, Kramer *et al*. 133, and Skubal *et al*. 134, respectively, under CC BY 4.0. Panel B(i) was created by the authors in BioRender.com based on Maringanti *et al*. 121 (TAO, T., 2026; https://BioRender.com/jypgj5h).

**Figure 5:**
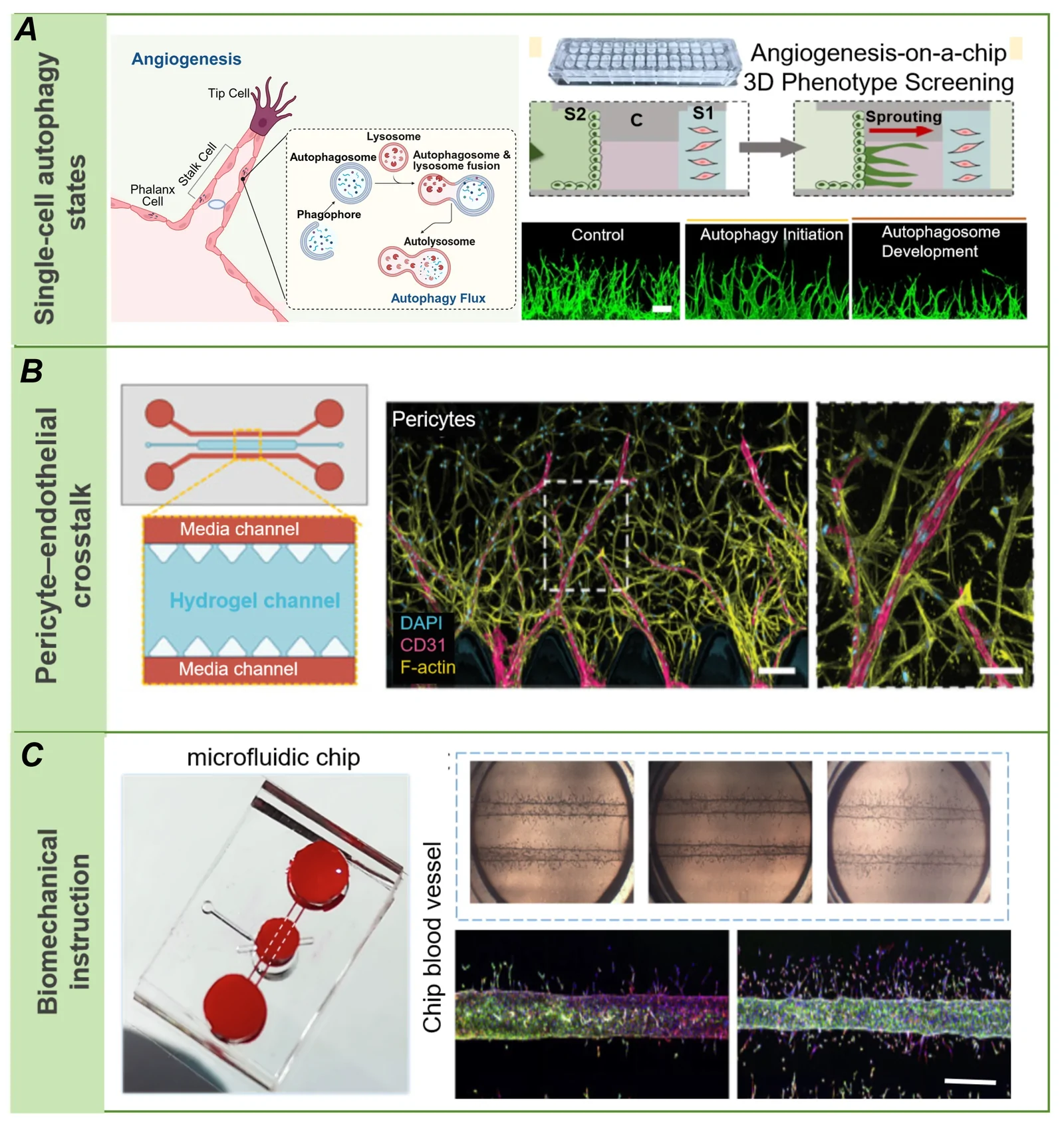
** Representative AoC-based strategies for mechanistic interrogation of angiogenic regulation.** (A) Single-cell autophagy states. Left: Schematic of sprouting angiogenesis showing tip, stalk, and phalanx endothelial cells with spatially heterogeneous autophagy activity, including autophagosome formation, maturation, and lysosomal fusion, reflecting cell-state-specific metabolic demands. Right: AoC enables three-dimensional phenotypic screening of endothelial sprouting under controlled conditions. Representative images show that autophagy modulation alters sprout density and morphology, highlighting the value of microfluidics and single-cell analysis for investigating metabolic regulation of angiogenesis, scale bar: 200 μm. (B) Microfluidic analysis of pericyte-mediated angiogenesis, showing enhanced vascularization through an IL-6-dependent mechanism, scale bar: 200 µm (left) and 100 µm (right). (C) Biomechanical regulation. One configuration examines ECM stiffness-dependent sprouting, whereas a separate configuration examines cyclic-stretch effects on vasculogenesis-like network formation, scale: 500μm. Panel A-left was created by the authors in BioRender.com based on Lee *et al*. 39 (TAO, T., 2026; https://BioRender.com/toepmif). Panels A-right, B, and C were adapted from Lee *et al*. 39, Gonzalez-Rubio *et al*. 108, and Ferrari *et al*. 147, respectively, under CC BY 4.0.

**Table 1:** Definitions and applications of AoC

Vascular process	Definition	Application in AoC models	Ref.
Angiogenesis	Formation of new vessels from pre-existing vasculature, usually involving endothelial activation, sprouting, lumen formation, remodeling, and perfusion.	Requires direct evidence of vessel-derived growth; perfusion or maturation alone is insufficient.	9, 14
Vasculogenesis	De novo organization of initially dispersed ECs into vascular networks; network assembly by mature ECs *in vitro* is more precisely described as vasculogenesis-like.	Generates the initial vascular bed; subsequent angiogenesis requires new growth from that bed.	9, 15
Vascularization	Broad formation of vascular structures within tissues, organoids, or engineered constructs by angiogenesis, vasculogenesis, endothelialization, bioprinting, or ingrowth.	Included when vascularization informs sprouting, remodeling, perfusion, barrier formation, maturation, or disease modeling in AoC systems.	7, 16
Endothelialization	Formation of an endothelial lining on a predefined channel, scaffold, membrane, or blood-contacting surface.	Not angiogenesis by itself. Included when the endothelialized structure serves as a parent vessel or functional vascular interface.	15-16
Vascular maturation	Stabilization of nascent vessels through pericyte or SMC coverage, basement-membrane deposition, junctional organization, barrier regulation, and sustained perfusion.	Used as a validation dimension for AoC models beyond morphology-based vessel formation.	9, 17

**AoC**: angiogenesis-on-a-chip; **EC**: endothelial cell; **SMC**: smooth muscle cell.

**Table 2:** Engineering strategies for AoC construction

Strategy	Vascular process and AoC boundary	Key parameters and validation endpoints	Applications	Limitations	Ref.
Vascular patterning	Models sprouting angiogenesis when endothelial cells grow from a predefined parent vessel into a 3D matrix and form new lumens or connections. Static channel lining alone is an endothelialized vessel or barrier model.	Report lumen diameter, EC–matrix interface, hydrogel width, flow rate, WSS, matrix stiffness, and gradient design; Validate lumen continuity, perfusion, permeability, endothelial junctions, pericyte or SMC coverage, and flow or drug response.	Defined geometry; Controlled flow and gradients; Barrier and antiangiogenic drug assays	Over-constrained vessel shape; Limited spontaneous remodeling; Sensitive to leakage and incomplete endothelialization	13, 46-49
Self-assembled vascular networks	Dispersed endothelial cells undergo de novo assembly in ECM, a process resembling vasculogenesis. Subsequent angiogenesis requires directly tracking growth originating from pre-existing vessels; anastomosis, perfusion, and remodeling are functional or maturation endpoints.	Report initial cell configuration, EC/support-cell source, matrix, and flow; Separately assess vessel origin, lumenization, connectivity, perfusion, permeability, mural-cell coverage, and stability	Capillary-bed generation; Organ-specific vascularization; Subsequent vessel-derived sprouting assays and post-formation remodeling studies	The topology is stochastic, and local shear stress is heterogeneously distributed; Lower reproducibility	50-53
3D bioprinting /Stem-cell co-differentiation	Printed or stem-cell-derived vascularized constructs; AoC-relevant only after chip integration, perfusion, and functional validation.	Report bioink properties, printing shear, crosslinking method, resolution, channel size, cell ratio, induced pluripotent stem cell (iPSC) differentiation protocol, perfusion integration, maturation time, and batch-to-batch consistency; Validate endothelial viability, lumen continuity, stable perfusion, permeability, junctional integrity, pericyte or SMC coverage, and tissue-specific maturation markers.	Spatially programmed multicellular constructs; Scalable vascularized OoC production; Stem-cell-derived and personalized models	Printing resolution limits; Cell injury and bioink constraints; iPSC immaturity and batch variability	14, 54-57

**EC**: endothelial cell; **iPSC**: induced pluripotent stem cell; **SMC**: smooth muscle cell; **WSS**: wall shear stress.

**Table 3:** Function-oriented design requirements for AoC systems

Design domain	Elements	Typical AoC	Parameters to report	Main applications	Limitations and validation
Materials	PDMS substrate	PDMS endothelialized chip; PDMS sprouting chip	Mixing ratio; Curing; Bonding; Plasma conditions and purpose; treatment-to-use interval; Coating; Geometry	Device optimization; Live imaging; Sprouting and perfusion	Check hydrophobic recovery, solvent swelling, residual-oligomer leaching, gas/evaporation control, and hydrophobic-compound loss by sorption
	Rigid and lower-sorption substrates	COC or COP chip; PMMA, PC, or PS chip; Glass or silicon chip; Validated printed-polymer chip	Material grade; Fabrication; Bonding; Coating; Oxygen control	Drug testing; Imaging; Permeability and barrier assays	Lower gas exchange and high rigidity; Confirm coating quality and drug recovery for drug assays
	Natural ECM hydrogel	Collagen I; Fibrin; Collagen–fibrin; Matrigel-containing matrix	Source and lot; Concentration; Gelation; Stiffness; Permeability; Geometry	Sprouting; Invasion; Lumenization and anastomosis	Matrix concentration simultaneously alters stiffness, ligand density, pore structure, and transport; Matrigel is batch variable
	Tunable or tissue-derived matrix	GelMA; PEG or PEG-norbornene; HA; Engineered protein; dECM matrix	Crosslinking; Ligands; Degradability; Stiffness; Viscoelasticity; Batch quality	Matrix-controlled angiogenesis; Bioprinting; Vessel stabilization	Assess ligand functionalization, light or initiator toxicity, uneven crosslinking, and dECM variability
Physical cues	Luminal flow and WSS	Endothelialized channel; Perfusable vascular network	Flow rate; WSS; Viscosity; Diameter; Pulsatility; Duration	Endothelial alignment under flow; Barrier integrity; Stable perfusion	WSS can vary across channels and networks; Validate flow with CFD, tracers, or particle tracking
	Interstitial flow and solute transport	Hydrogel pressure-gradient model; Source-sink gradient model	Pressure gradient; Velocity; Permeability; Gradient stability; Flow direction	Directional sprouting; Chemotaxis; Vessel connection	Leakage, gel compaction, evaporation, or changing permeability can distort gradients
	Matrix and pressure mechanics	Stiffness-tuned gel; Hydrostatic-pressure model; Cyclic-stretch chip; Deformable-membrane chip	Stiffness; Viscoelasticity; Pressure; Strain amplitude; Frequency; Wall mechanics	Matrix stiffness response; Pressure response; Large-vessel or aneurysm-like modeling	Match mechanics to the target vascular bed; Avoid applying stretch or pressure when not biologically relevant
	Vascular geometry	Straight endothelialized lumen; Branched vascular network; Multilayer vascular wall; Capillary-like vascular bed	Diameter; Cross-section; Branch hierarchy; Mural-cell layout; Perfusion route	Capillary sprouting; Microvascular-bed modeling; Arteriole-like and venule-like modeling	Geometry alone does not prove function; Verify lumen formation, perfusion, and barrier integrity
Biological modules	Vascular cell composition	EC-pericyte co-culture; EC-SMC co-culture; EC-fibroblast or stromal co-culture	Cell source; Donor; Passage; Ratio; Spatial layout; Culture sequence	Pericyte or SMC coverage; Vessel stabilization; Organ-specific angiogenesis	Cell source and seeding order affect stability; Report donor, passage, and cell ratio
	Angiogenic signaling	VEGF-A/VEGFR2 gradient model; DLL4-Notch tip-stalk model; Ang-1/Tie2 maturation model; PDGF-BB/PDGFRβ mural-cell recruitment model	Concentration; Spatial gradient; Exposure duration; Timing; Cell source	Tip-stalk patterning; Sprouting; Mural recruitment; Vessel stabilization	Avoid adding growth factors generically; Match dose, timing, and location to the model aim
	Immune and lymphatic module	Macrophage-vascular model; Neutrophil recruitment model; Lymphatic endothelial chip	Immune-cell source and activation state; Cytokines; Spatial placement; VEGF-C-dependent lymphangiogenic signaling; lymphatic markers	Inflammatory angiogenesis; Tumor angiogenesis; Lymphangiogenesis	Add these modules only when needed; Distinguish blood vessels from lymphatic vessels
Analytical validation	Process-specific and computational validation	Perfusion assay; Permeability assay; Marker imaging; AI segmentation; CFD or oxygen modeling	Predefined endpoints; Image-analysis thresholds; Model boundary conditions; Validation method; Reproducibility	Perfusion; leakage; network features; shear or oxygen maps; phenotype classification	Report algorithms, validation metrics, manual correction, and reproducibility across chip types

**AI**: artificial intelligence; **Ang-1**: angiopoietin-1; **DLL4**: Delta-like ligand 4; **ECM**: extracellular matrix; **Tie2**: tyrosine kinase with immunoglobulin-like and EGF-like domains 2; **VEGF-A**: vascular endothelial growth factor A; **VEGF-C**: vascular endothelial growth factor C; **AoC**: angiogenesis-on-a-chip; **CFD**: computational fluid dynamics; **COC**: cyclic olefin copolymer; **COP**: cyclic olefin polymer; **dECM**: decellularized extracellular matrix; **EC**: endothelial cell; **GelMA**: gelatin methacryloyl; **HA**: hyaluronic acid; **PC**: polycarbonate;** PDGF-BB**: platelet-derived growth factor-BB; **PDGFRβ**: platelet-derived growth factor receptor β; **PDMS**: polydimethylsiloxane; **PEG**: polyethylene glycol; **PMMA**: polymethyl methacrylate; **PS**: polystyrene; **SMC**: smooth muscle cell; **VEGF**: vascular endothelial growth factor; **VEGFR2**: vascular endothelial growth factor receptor 2; **WSS**: wall shear stress.

## Abbreviations

AI: artificial intelligence
ALK5: activin receptor-like kinase 5
Ang-1: angiopoietin-1
AoC: angiogenesis-on-a-chip
CAR-T: chimeric antigen receptor T cell
CFD: computational fluid dynamics
COC: cyclic olefin copolymer
COP: cyclic olefin polymer
CoU: context of use
dECM: decellularized extracellular matrix
DLL4: Delta-like ligand 4
DLP: digital light processing
EC: endothelial cell
ECFC: endothelial colony-forming cell
ECM: extracellular matrix
FGF-2: fibroblast growth factor 2
GelMA: gelatin methacryloyl
HA: hyaluronic acid
HES1: hes family bHLH transcription factor 1
HEY1: hes-related family bHLH transcription factor with YRPW motif 1
HIF: hypoxia-inducible factor
hPSC: human pluripotent stem cell
HUVEC: human umbilical vein endothelial cell
ICAM-1: intercellular adhesion molecule 1
IL-6: interleukin-6
iPSC: induced pluripotent stem cell
MMP: matrix metalloproteinase
NG2: neuron-glial antigen 2
OoC: organ-on-a-chip
PBPK: physiologically based pharmacokinetic
PC: polycarbonate
PDGF-BB: platelet-derived growth factor-BB
PDGFRβ: platelet-derived growth factor receptor β
PDMS: polydimethylsiloxane
PEG: polyethylene glycol
PMMA: polymethyl methacrylate
PS: polystyrene
PSMA: prostate-specific membrane antigen
SMC: smooth muscle cell
TGF-β: transforming growth factor-β
Tie2: tyrosine kinase with immunoglobulin-like and EGF-like domains 2
VCAM-1: vascular cell adhesion molecule 1
VEGF: vascular endothelial growth factor
VEGFR2: vascular endothelial growth factor receptor 2
WSS: wall shear stress
ZO-1: zonula occludens-1
α-SMA: α-smooth muscle actin
